# OmniCellTOSG: The First Cell Text-Omic Signaling Graphs Dataset for Graph Language Foundation Modeling

**DOI:** 10.21203/rs.3.rs-8774770/v1

**Published:** 2026-02-17

**Authors:** Heming Zhang, Tim Xu, Dekang Cao, Shunning Liang, Guntaas Shergill, Nicholas Hadas, Lars Schimmelpfennig, Levi Kaster, Di Huang, Guangfu Li, S. Peter Goedegebuure, David DeNardo, Li Ding, Ryan C. Fields, J Philip Miller, Pirooz Eghtesady, Carlos Cruchaga, William Buchser, Jonathan Cooper, Marco Sardiello, Patricia Dickson, Yixin Chen, Michael Province, Philip Payne, Fuhai Li

**Affiliations:** 1 Institute for Informatics, Data Science and Biostatistics, Washington University in St. Louis, 4444 Forest Park Ave., Saint Louis, 63108, MO, USA.; 2 Department of Computer Science, Washington University in St. Louis, 1 Brookings Dr., Saint Louis, 63130, MO, USA.; 3 NeuroGenomics and Informatics Center, Washington University in St. Louis, 4444 Forest Park Ave., Saint Louis, 63108, MO, USA.; 4 Department of Genetics, Washington University in St. Louis, 4515 McKinley Ave., Saint Louis, 63110, MO, USA.; 5 Department of Surgery, Washington University in St. Louis, 4444 Forest Park Ave., Saint Louis, 63110, MO, USA.; 6 Siteman Cancer Center, Washington University in St. Louis, 4444 Forest Park Ave., Saint Louis, 63110, MO, USA.; 7 Department of Medicine, Washington University in St. Louis, 4444 Forest Park Ave., Saint Louis, 63110, MO, USA.; 8 Department of Pediatrics, Washington University in St. Louis, 4444 Forest Park Ave., Saint Louis, 63110, MO, USA.; 9 Department of Surgery, University of Connecticut, 263 Farmington Ave., Farmington, 06032, CT, USA.

**Keywords:** Foundation Models, Text-Omic Signaling Graph, Graph Language Foundation Models, Single Cell

## Abstract

With the rapid growth of large-scale single-cell omic datasets, omic foundation models (FMs) have emerged as powerful tools for advancing research in life sciences and precision medicine. However, most existing omic FMs rely primarily on numerical transcriptomic data by sorting genes as sequences, while lacking explicit integration of biomedical prior knowledge and signaling interactions that are critical for scientific discovery. Here, we introduce the Text-Omic Signaling Graph (TOSG), a novel data structure that unifies human-interpretable biomedical textual knowledge, quantitative omic data, and signaling network information. Using this framework, we construct OmniCellTOSG, a large-scale resource comprising approximately half million meta-cell TOSGs derived from around 80 million single-cell and single-nucleus RNA-seq profiles across organs and diseases. We further develop CellTOSG-FM, a multimodal graph language FM, to jointly analyze textual, omic and signaling network context. Across diverse downstream tasks, CellTOSG-FM outperforms existing omic FMs, and provides interpretable insights into disease-associated targets and signaling pathways.

## 1 Main

The human organism comprises ~37.2 trillion cells that arise from a single zygote and share a common genome, yet acquire specialized identities through context-dependent signaling. Such signaling is orchestrated by transcriptional programs, protein abundance and modification, and protein–protein interactions, and is further conditioned by age, sex, diet, environmental exposures, and disease state. Despite decades of discovery, major gaps persist: system-level, cell-resolved inventories of signaling entities and edges; quantitative models of network rewiring across lifespan and pathology; principled detection of disease-relevant subpopulations and their intercellular crosstalk; and actionable strategies to perturb these networks to prevent or reverse disease. Single-cell and single-nucleus RNA sequencing (sc/snRNA-seq) now provide transcriptome-wide measurements at cellular resolution, enabling delineation of cell types/subtypes in healthy and diseased tissues and the study of signaling interactions within niches or microenvironments. Large-scale efforts, such as the CZ CELLxGENE [1, 2], the Human Cell Atlas [3], the Brain Cell Atlas [4], and numerous disease-focused studies [5, 6], have generated hundreds of millions of profiles that support systematic interrogation of signaling. These resources make it feasible to ask not only which genes are active, but how groups of genes/proteins with distinct abundance levels coordinate to realize specific biological functions across diverse cellular contexts.

Currently, foundation models trained via self-supervised objectives have transformed representation learning. Most foundation single-cell models operate on expression vectors and typically do not incorporate explicit pathway structure, including SCimilarity [7], GeneFormer [8], scGPT [9], scFoundation [10], and scCello [11]. Despite recent progress, these approaches generally omit explicit modeling of signaling graphs, limiting inference of dysfunctional pathways and decoding of graph-structured signaling patterns across conditions. Recent progress on training graph foundation models has explored masked reconstruction objectives within masked graph modeling, with node-masking methods such as GraphMAE [12] as representative examples. Systematic analysis suggests that masking edges rather than nodes yields stronger performance on structure-sensitive tasks, including link prediction and topology recovery, and better captures relational patterns [13]. This is particularly salient for cellular signaling, where functional meaning arises from interaction topology rather than isolated node attributes. Interpreting these structure-dependent mechanisms typically requires both biomedical prior knowledge and topological information, yet existing models remain limited in both aspects: LLMs often struggle with domain-specific biomedical reasoning and may produce hallucinated or unreliable outputs [14], whereas GNNs can be limited in modeling complex higher-order relational structures [15, 16]. In addition to these limitations, purely numeric omicbased foundation models typically treat molecular measurements as isolated features and rarely incorporate human-interpretable textual biomedical priors and signaling-network context, which limits mechanistic interpretability and hypothesis-driven discovery. Prior work suggests that integrating biologically grounded knowledge graphs with quantitative omic features can improve predictive accuracy [17] and strengthen mechanistic reasoning by explicitly capturing cellular interactions [18]. Collectively, these challenges motivate the development of a unified representation that jointly incorporates textual priors, omic evidence, and signaling topology.

In this study, for the first time, we introduce (i) **Text–Omic Signaling Graphs (TOSGs)**, a novel data format that unifies textual biological priors (e.g., gene/protein functions, mechanisms) with numerical omic data to support graph-based interpretation of cell signaling; (ii) **OmniCellTOSG**, a large-scale biomedical AI resource aggregating approximately 80 million sc/snRNA-seq profiles across tissues, cell types, diseases, ages, sexes, and related attributes, providing a comprehensive data foundation to support the development and benchmarking of next-generation AI foundation models for scientific discovery at an expert level; and (iii) **CellTOSG Foundation Model (CellTOSG-FM)**, a multi-modal graph language foundation model that couples textual biological priors and numerical omic evidence with topological signaling network over TOSGs and cross-modalities encoders to augment graph representation learning and to support downstream tasks, including cell-type annotation, cell attribute classification, and signaling inference with interpretable graph rationales (iv) All the data and code are publicly accessible. OmniCellTOSG dataset is accessible at: huggingface.co/datasets/FuhaiLiAiLab/OmniCellTOSG_Dataset and CellTOSG-FM code is available at: github.com/FuhaiLiAiLab/OmniCellTOSG

## 2 Results

### 2.1 OmniCellTOSG ecosystem overview

**Fig. 1:**
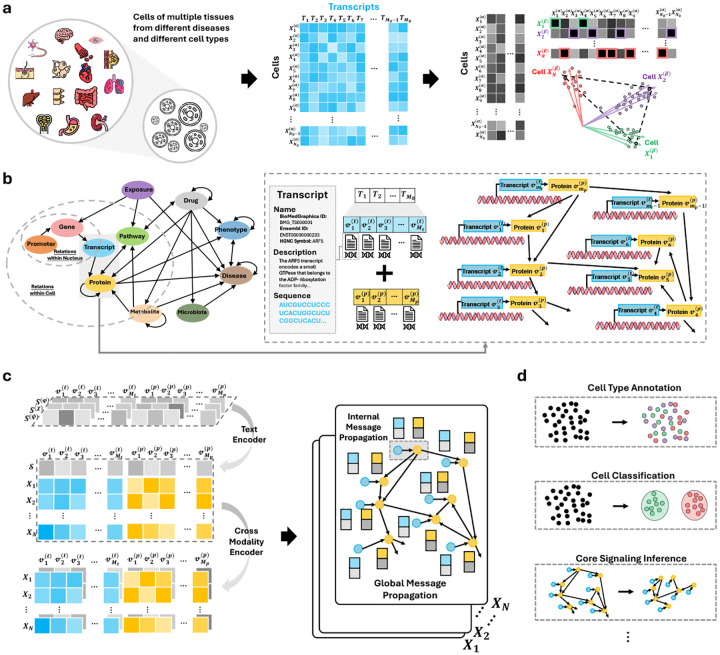
OmniCellTOSG: data construction and foundation model pipeline. **(a)** Millions of sc/snRNA-seq profiles from diverse tissues, diseases, and cell types (h5ad) are integrated; transcript matrices are extracted from h5ad files and $N_{0}$ cells are transformed into N meta-cells via archetypal analysis. **(b)** Knowledge-grounded graph assembly: transcript and its downstream protein entities are aligned to the BioMedGraphica knowledge base $\left(M=M_{t}+M_{p}\right)$ to form Text–Omic Signaling Graphs (TOSGs) with both matched and virtual entities, capturing nucleus-level and intra-cell relations. **(c)** Cross-modal representation learning: a language-modality encoder embeds biological priors (entity names, descriptions, sequences) and is fused with omic features; message passing operates within cells (internal) and across the TOSG (global) to yield unified representations for pretraining and downstream tasks. **(d)** Example downstream tasks enabled by the pretrained model: cell-type annotation, disease classification, core-signaling inference, etc.

We present an integrated ecosystem that couples a large, knowledge-grounded single-cell resource with reproducible tooling and a multi-modal graph language foundation model (see Figure 1). **OmniCellTOSG** aggregates approximately $N_{0}(N_{0}≃80$ million) million single-cell and single-nucleus RNA sequencing profiles into N(N≃0.5 million) representative meta-cells using the archetypal analysis framework implemented in SEACells [19]. This aggregation preserves biological diversity across tissues, diseases, age groups, and experimental conditions (Figure 1a). Based on the resulting meta-cell transcriptomic expression matrices, we next construct **Text–Omic Signaling Graphs (TOSGs)** by mapping the $M_{0}$ transcriptomic entities to $M_{t}$ transcript nodes and introducing $M_{p}$ corresponding protein nodes, with edges linking transcript entities to their associated proteins. The resulting graph therefore contains a total of M nodes, where $M=M_{t}+M_{p}$ (see Section 4.1 for details). These graphs integrate quantitative omics measurements with curated biological knowledge from BioMedGraphica on the vertex set 𝒱 (see Section 4.2). Formally, the overall entity set is defined as $𝒱=\left\{{𝒱}^{\left(t\right)},{𝒱}^{\left(p\right)}\right\}$, with $|𝒱|=\left|{𝒱}^{\left(t\right)}\right|+\left|{𝒱}^{\left(p\right)}\right|=M_{t}+M_{p}=M$, thereby linking molecular signals with established biomedical prior knowledge. TOSG supports both matched and virtual entities and records intra-cell and nucleus-level relations, enabling graph-structured signaling beyond expression vectors alone (see Figure 1b). For each entity, numerical omics features are used to form a unified representation. Transcript nodes contain measured transcriptomic expression values, while virtual protein nodes are zero-initialized because no proteomic measurements are available. These features are assembled into a global omics feature matrix 𝒳. In addition to the transcriptomic and virtual proteomic features, we incorporate an auxiliary textual annotation dataset, $𝒮=\left\{S^{\left(\varphi \right)},S^{\left(\chi \right)},S^{\left(\psi \right)}\right\}$, which provides complementary semantic information of entity name, description and biosequences (i.e., RNA sequences and protein sequences) for each node (see Section 4.2 and Figure 1c for details). Moreover, CellTOSG_Loader provides a NumPy-ready query–load–balance pipeline that constructs stratified, unbiased cohorts across user-specified facets (cell type, tissue, disease, data source, age/sex, etc.), mitigates class imbalance for pretraining and downstream evaluation. In addition, **CellTOSG-FM** integrates textual biological priors and omic features through cross-modal encoders. The graph encoder then performs message propagation within cells and across TOSGs to produce fused representations that enable cell-type annotation, disease classification, signaling-pathway inference, and drug-response prediction, together with interpretable subgraph rationales (see Section 4.4.1 and Figure 1c for details). Collectively, these components constitute a scalable, mechanism-focused framework that standardizes data ingestion, supports fair and reproducible experimentation, and facilitates knowledge-augmented modeling of cellular signaling at scale. The subsequent sections detail each component.

### 2.2 OmniCellTOSG Dataset

**Fig. 2:**
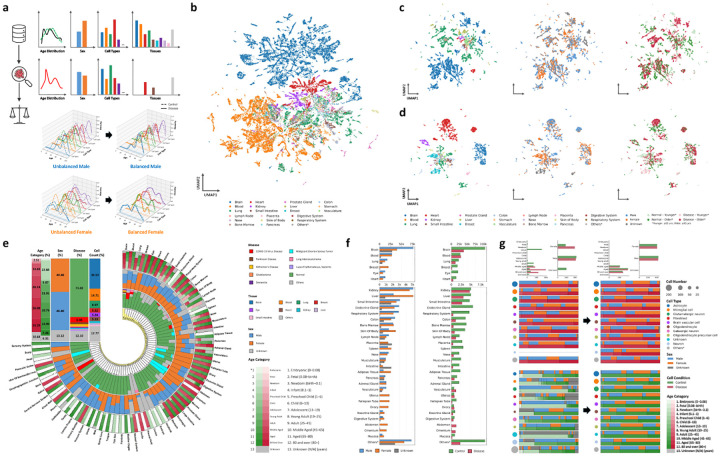
OmniCellTOSG dataset composition and balancing demo. **(a)** Three-step balancing workflow: first, the raw dataset and its age/sex/cell-type/tissue distributions; second, the retrieved disease-specific distribution used as the target; third, the balancing step and the resulting distributions, such as density overlays for male and female subsets. **(b)** UMAP embedding of representative sampled cells colored by types of tissue of origin, highlighting comprehensive inclusion. **(c-d)** UMAPs of macrophage cells (upper panel) and fibroblast cells (lower panel) colored by tissue of origin, sex, age groups and disease conditions. **(e)** Global composition of the integrated cohort shown as concentric summaries stratified by disease, tissue, sex, age group, and cell counts. **(f)** Exact cell counts per tissue, stratified by sex and condition (control vs. disease). **(g)** Alzheimer’s disease case study: before/after balancing comparisons of the retrieved-and-loaded dataset using stacked bars of cell-type composition, with overlays indicating cell number, sex proportion, condition, and age category proportion.

We introduce **OmniCellTOSG**, a large-scale single-cell resource that integrates transcriptomic profiles from CellxGene [1, 2], the Brain Cell Atlas [4], GEO [20], Single Cell Portal [21], and the Human Cell Atlas [22], paired with rich textual annotations spanning diverse tissues and disease states. Starting from 79,195,364 cells, we performed rigorous preprocessing—including quality control, normalization, and harmonization of organ/tissue and disease labels. Cells were aggregated into meta-cells using SEACells [19] and coupled with prior biological knowledge from BioMedGraphica [23] to assemble Text–Omic Signaling Graphs (TOSGs), yielding a curated set of 395,317 meta-cells. Attribute sets were standardized to the Cell Ontology [24] (766 cell types across 65 tissues) and disease annotations were mapped to the BioMedGraphica nomenclature (140 disease states), with remaining fields normalized for retrieval metadata. Following profile harmonization, transcriptomic data were linked to transcript entities and their downstream protein counterparts in BioMedGraphica to construct TOSGs with both matched and virtual entities, capturing nucleus-level and intra-cell relationships. In total, the graph comprises 533,458 entities and 16,637,405 relations (152,585 internal interactions and 16,484,820 protein–protein interactions). A high-level overview of the integrated resource, OmniCellTOSG, is presented in Figure 2b, and the full data-processing methodology is detailed in Section 4.1.

To ensure reproducibility and a model-ready data format, we release CellTOSG_Loader (Section 4.3), which transforms user-specified parameters into executable queries, loads matched subsets, and performs stratified cohort balancing to mitigate confounding. The loader further applies platform-aware and sc/snRNA-aware batch correction via ComBat–seq [25, 26] to reduce variance across data sources and profiling platforms (Figure S1). As an illustrative use case, for an Alzheimer’s disease (AD) versus control comparison, the loader matches the control cohort to the AD distribution over sex, age categories, and cell-type composition. Figure 2a outlines the balancing workflow, and Figure 2g shows before/after distributions for AD versus matched normals.

### 2.3 CellTOSG-FM Construction and Pretraining

**Fig. 3:**
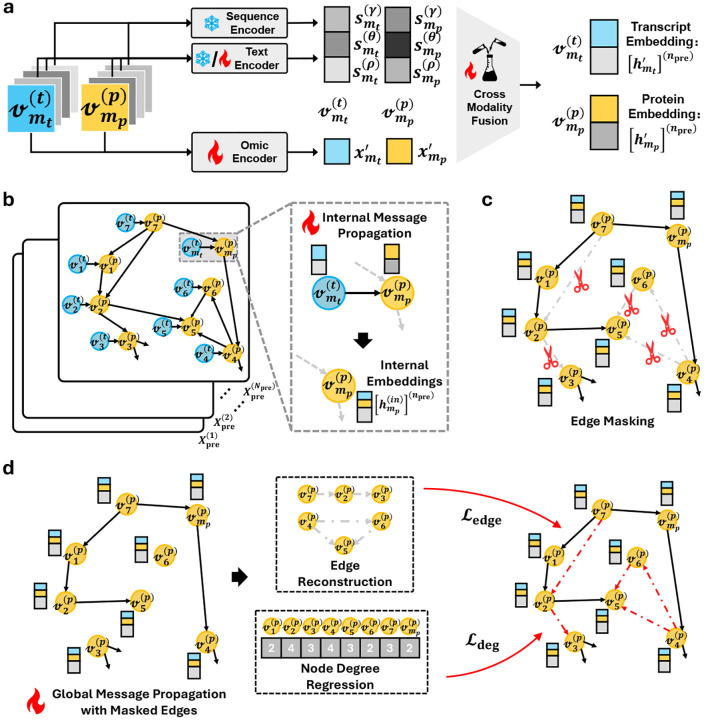
Overview of CellTOSG-FM Pretraining. **(a)** Cross-modal representation learning: a text encoder and an omic encoder are fused to embed nodes. **(b)** Messages propagate within nucleus (internal) and across the TOSG (global) to encode fused biological and textual context for pretraining model. **(c)** Mask edges by random sampling. **(d)** Global message propagation via masked edges. Edge reconstruction and node-degree prediction are used as pretraining objectives, optimized via edge-reconstruction loss $\left({ℒ}_{\text{edge}}\right)$ and node degree regression loss ${(ℒ}_{\text{deg}})$.

We pretrained the model, $f_{\text{pre}}(\cdot )$, in a self-supervised manner on a subset ${𝒳}_{\text{pre}}\subseteq 𝒳$, without using any metadata from the attribute set 𝒜. As shown in Figure 3, sequence information is encoded with DNA-GPT [27] for RNA sequences (thymine T substituted by uracil U) and ProtGPT2 [28] for protein sequences. These encoders provide high-capacity, transferable representations learned from large-scale genomic and proteomic corpora, and have demonstrated strong generalization on diverse downstream biological tasks. Because transcript and protein entities in BioMedGraphica are stable and reused across samples, we freeze the sequence encoders to eliminate redundant computation, reduce overfitting, and ensure reproducibility across runs; users may substitute alternative sequence language models if desired. A trainable omic encoder then maps numerical omic measurements into the same latent space, and a cross-modality encoder integrates the textual/sequence priors with the quantitative omic evidence to form unified entity representations (Figure 3a). This design allows the model to leverage complementary information sources, including semantics and biochemistry from sequences and context-specific variation from omics, within a single representational framework.

Furthermore, we incorporate topological structure by encoding the latent representations with graph encoders. Concretely, messages are propagated to protein entities in two coupled stages: an internal message-passing step that aggregates signals within transcript–protein pairs at the nucleus level, followed by a global propagation step that diffuses information within the cell via the protein–protein interaction topology. The latter is trained with stochastically masked edges (Figure 3b–c), optimizing a joint objective that combines edge reconstruction with a degree-regularization term to calibrate node centrality. Consistent, monotonic reductions in the training objective are observed, and held-out edge recovery improves throughout optimization; the degree-oriented auxiliary term further sharpens hub–periphery structure and stabilizes learning (Figure 3d). Using an edge-masking ratio of 10^−5^ for the self-supervised objective [13], the model reconstructs about 80% of masked edges, and attains an AUC near 0.85 when it converges (see Figure S2). Notably, these outcomes are achieved when pretraining on 5% of OmniCellTOSG for ${𝒳}_{\text{pre}}$, underscoring the sample efficiency of the approach and its suitability under limited pretraining budgets. Additional details of the CellTOSG-FM pretraining protocol are provided in Section 4.4.

### 2.4 Downstream Tasks Based on CellTOSG-FM

**Fig. 4:**
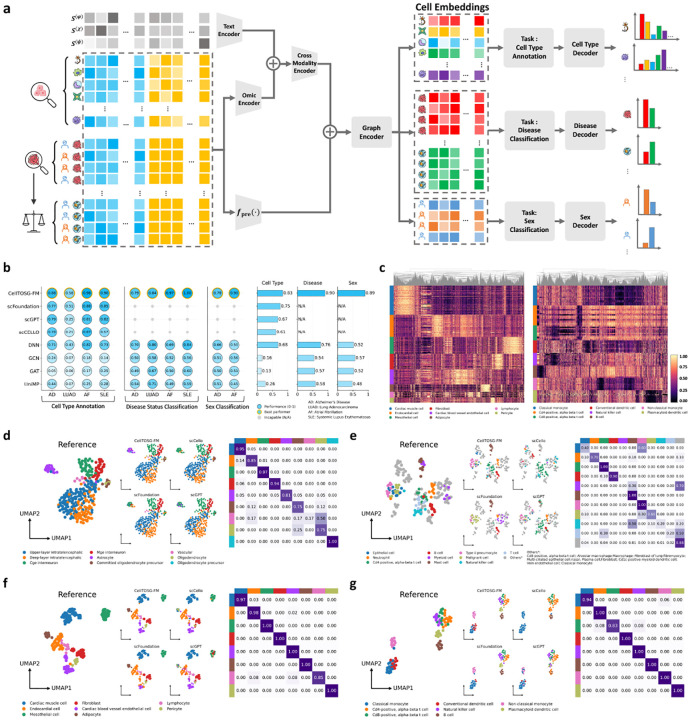
Experimental results of CellTOSG-FM performance on cell type annotation and cell classification task. **(a)** An illustration of the cell type annotation and cell classification model based on CellTOSG-FM. **(b)** Performances on cell type annotations and cell condition classifications (disease vs. normal) on four different diseases (AD, LUAD, AF and SLE), and cell sex classification (male vs. female) on AD and AF. **(c)** Cell embeddings learned by the foundation model for cell type annotation in AF and SLE datasets, showing clear separation of cell identities in latent space prior to the classification decoder. **(d-g)** Comparison of cell type annotations performances on AD, LUAD, AF and SLE. on UMAP between OmniCellTOSG ground truth and predictions from four models (CellTOSG-FM, scCello, scFoundation, and scGPT). Confusion matrices report the ten most abundant cell types in the sampled dataset, with remaining types grouped as “Others” for visualization while retained as distinct classes for evaluation.

After pretraining the foundation model CellTOSG-FM, we preserve the model architecture $f_{\text{pre}}(\cdot )$ together with its pretrained parameters $\omega_{\text{pre}}$ as a transferable initialization for downstream adaptation. For each downstream task, we employ the CellTOSG_Loader to extract and organize the task-specific datasets for fine-tuning. In our downstream tasks, lung adenocarcinoma (LUAD), atrial fibrillation (AF) and systemic lupus erythematosus (SLE) cohorts are sourced from OmniCellTOSG. We additionally evaluate on an external Alzheimer’s disease (AD) single-cell cohort from the GSE129308 project, which is available across both CELLxGENE and Brain Cell Atlas and was held out from OmniCellTOSG to enable an independent evaluation (see Section 5.4 for processing details). In general, the extracted samples/cells are first embedded using the pretrained foundation model $f_{\text{pre}}(\cdot )$, together with the downstream omic and text encoders, to generate integrated feature representations. Subsequently, gene-level information for each sample/cell is projected into a latent embedding space, denoted as ${𝒵}^{\left(\tau \right)}$, which serves as the input for task-specific predictors and facilitates efficient adaptation across diverse biological tasks (see Figure 4a and Section 4.5 for details).

#### 2.4.1 CellTOSG-FM Improves Cell Type Annotation

Using cell-specific embeddings generated from CellTOSG-FM, downstream omic and text encoder and downstream message propagation via GNN layers, the downstream cell-type decoder will be applied to predict the cell-type (Figure 4a), and we evaluated annotation performance here on four disease cohorts (AD, LUAD, SLE, and AF). For each cohort, CellTOSG_Loader was used for cohorts sourced from OmniCellTOSG, while an analogous pipeline was applied to the external AD cohort, to construct balanced downstream datasets by matching the empirical distributions over cell types, thereby mitigating class-imbalance effects. To ensure consistent and comparable evaluation across tasks while reducing computational overhead, we further subsampled approximately 1,000 meta-cells for each task-specific dataset. To avoid potential data leakage, samples were partitioned into training and testing sets using donor identity as the splitting criterion, thereby ensuring donor-level independence between the two datasets. The resulting splits typically allocated approximately 20–40% of samples to the test datasets.

Across all four cohorts, CellTOSG-FM matched or exceeded strong baselines (including DNN [29, 30], GCN [31], GAT [32], UniMP [33], scGPT, scFoundation, and scCELLO) with consistent gains in every disease (see Figure 4b and Table S4 for overall performances and Figure 4d–g for more details). These improvements indicate that integrating biological textual priors with numerical omic evidence via a cross-modality encoder, together with knowledge-graph–grounded topology in the graph encoder, yields representations that transfer robustly to disease-specific annotation without task-specific tuning.

Heatmaps derived from the learned cell representations demonstrate clearer block structure and well separated manifold on cell types. Figure 4c illustrates the latent embedding spaces for AF and SLE. After restricting the analysis to the 5,000 highest-variance genes and arranging cells by cell type, distinct cell populations occupy clearly separated regions of the latent space in both datasets, demonstrating the effectiveness of CellTOSG-FM in capturing cell-type-specific differences. UMAP projections show that CellTOSG-FM produces compact, well-separated clusters whose boundaries closely align with reference labels, whereas alternative methods exhibit fragmented clusters and label mixing for several closely related types (Figure 4d–g). The accompanying confusion matrices display stronger diagonal dominance and fewer systematic off-diagonal errors for CellTOSG-FM. Collectively, these analyses demonstrate that the fused text–omic, graph-aware embeddings produced by CellTOSG-FM deliver higher annotation accuracy and cleaner class separability across diverse disease contexts than single-modality or topology-agnostic baselines.

#### 2.4.2 CellTOSG-FM Enhances Cell Classification Accuracy

We extracted disease-specific datasets using CellTOSG_Loader for OmniCellTOSG cohorts and applying an analogous pipeline to the external AD cohort, balancing cohorts by matching empirical cell-type distributions and thereby mitigating class-imbalance effects. To test whether the fused text–omic, graph-aware representations from CellTOSG-FM support accurate prediction of cell-level attributes, we trained task-specific decoders on top of cell embeddings (Figure 4a). Two evaluation settings were considered: disease status (disease vs. normal) in AD, LUAD, SLE and AF sampled dataset; sex classification (male vs. female) in AD and AF sampled dataset. Across all cohorts, CellTOSG-FM achieved the highest accuracies relative to strong baselines (GCN, GAT, DNN, UniMP) (Figure 4b and Tables S5–S6). These gains were obtained without task-specific architectural changes, indicating that integrating biological textual priors with numerical omic evidence, together with knowledge-graph–grounded topology, yields representations that generalize effectively to diverse attribute-prediction tasks.

To assess model robustness, we examined sex classification within AF dataset, stratified by age groups and major cell types (cardiac muscle cell, cardiac blood vessel endothelial cell, fibroblast cell, adipocyte cell, mesothelial cell, and others) (see Table S7 and Table S8). Heatmaps of the learned gene hyperspace embeddings, restricted to the 5,000 highest variance genes, reveal consistent separation patterns that remain evident when cells are stratified by age group and cell type (Figure S3). Collectively, these analyses indicate that CellTOSG-FM enables accurate and robust predictions across cohorts and biological strata, while the embedding spaces exhibit clear and stable group-wise separation, providing additional structural evidence of model robustness.

### 2.5 CellTOSG-FM Is Interpretable to Rank Targets and Signaling Networks

**Fig. 5:**
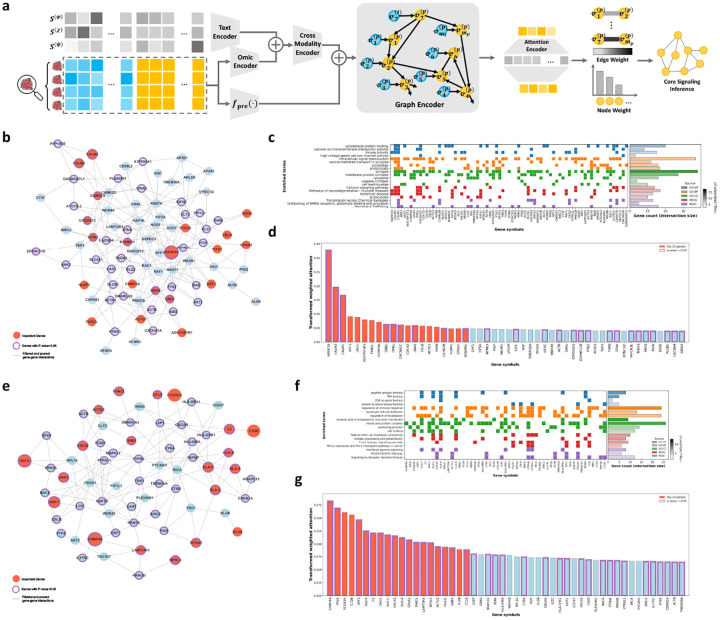
Target and signaling network inference using CellTOSG-FM. **(a)** Workflow for inferring disease-specific targets and signaling networks from retrieved samples. **(b–d)** Analysis results of AD: (b) inferred core disease-relevant signaling subgraph; (c) functional enrichment of genes in the AD core signaling subgraph; (d) ranked normalized gene-importance score distributions. **(e–g)** Analysis results of LUAD: (b) inferred core disease-relevant signaling subgraph; (f) functional enrichment of genes in the LUAD core signaling subgraph; (d) ranked normalized gene-importance score distributions. Purple nodes/bars (in b, d, e, g) mark the top 20 genes by importance; red outlines indicate genes with p value smaller than 0.05 by comparing disease and normal cells.

To illustrate interpretability, signaling targets and pathways were inferred per sample and summarized at the cohort level (Figure 5). Latent representations, ${𝒵}^{\left(\tau \right)}$, were translated into pairwise affinities between protein entities and then constrained to the protein–protein interaction (PPI) network, yielding edge weights that respect known biology. Node importance was computed by aggregating the strengths of incident edges and modulating by corresponding molecular signals, producing saliency scores that reflect both connectivity and activity. Cohort-level subgraphs were formed from top-ranked genes and pruned to eliminate spurious star-like branches while preserving informative connections, resulting in compact, connected networks aligned to each disease context (see Figure 5a and Section 4.5 for details).

For each sample, directed edge weights between BioMedGraphica nodes were first aggregated and mapped to their corresponding gene names. Edge weights, collected from the attention-based model, sharing the same source–target gene pairs were summed within each sample, reciprocal directions were averaged to obtain undirected edge weights, and edges from each individual sample within the same group were combined by aggregating weights of identical gene pairs across samples (Figure 5a). From the attention-based undirected graph, we derived node attention by averaging the attention weights of all edges incident to each node. In parallel, node expression value was derived by selecting, for each gene, the transcript index with the highest mean (min–max normalized) expression across samples, and group-wise average expressions and Mann–Whitney U p-values of comparing disease and control groups were then calculated for each node. Afterwards, the importance of nodes was ranked by using a node importance score [34] defined as the product of node attention and averaged expression of the corresponding group. Subsequently, the most important ξ=120 nodes per group were retained. Then, resulting signaling subgraph was refined by keeping only the largest connected component and pruning star-like leaf artifacts while preserving at least ϵ=3 leaves per node, preferentially retaining those with significant p-values or otherwise the highest-weight neighbors [35].

Fig 5a summarizes the pipeline from retrieved disease cohorts to targets and signaling networks subgraphs. Fig 5b–d show results for Alzheimer’s disease, where the inferred targets and signaling networks network recovers a compact set of connected signaling modules (red) with multiple statistically supported genes (purple outlines; p<0.05), and functional enrichment recapitulates key neurodegenerative processes (Figure 5c–d). Enrichment analysis highlights synapse-associated and cytoskeleton-related programs, including synapse and cytoskeletal protein binding, consistent with extensive evidence that early AD pathogenesis involves synaptic vulnerability and disrupted actin/cytoskeletal regulation [36–38]. Calcium-related molecular functions and pathways are also well represented, including calcium ion transmembrane transporter activity, high voltage-gated calcium channel activity, and Calcium signaling pathway, supporting the long-standing Ca^2+^ dysregulation framework in AD and implicating VGCC/NMDAR-linked signaling as a mechanistic contributor to synaptic failure and neurotoxicity [39–41]. Consistently, synaptic transmission terms (Transmission across Chemical Synapses and Unblocking of NMDA receptors, glutamate binding and activation) align with prior work linking aberrant glutamatergic/NMDAR signaling to AD synaptic dysfunction [42, 43]. Trafficking-related biology is strongly supported by enrichment of endocytosis, vesicle-mediated transport in synapse, Endocytosis, and Membrane Trafficking, supported by evidence that endocytosis and vesicle recycling defects are early and mechanistically relevant to AD [44, 45]. Moreover, enrichment of autophagy and lysosome aligns with reports of autophagy–lysosome dysfunction in AD [46, 47]. Finally, kinase-centric and signaling-related terms (kinase activity and intracellular signal transduction) are consistent with evidence that dysregulated neuronal signaling cascades contribute to AD progression [48, 49], suggesting that the inferred signaling pathways form a compact, connected network consistent with reference neurodegeneration/AD pathway frameworks.

Figure 5e–g show the inferred LUAD targets and signaling networks network and its functional enrichment. The enrichment results are dominated by antigen presentation and immune effector programs, with additional checkpoint and growth/angiogenesis signaling. Enrichment of antigen processing/presentation pathways and functional categories (e.g., peptide antigen binding, TAP binding, and Antigen processing and presentation) is consistent with established mechanisms by which altered HLA/APM function shapes tumor immune evasion and immunotherapy responsiveness in lung cancer [50, 51]. In parallel, enrichment for cytotoxic immune pathways (Natural killer cell mediated cytotoxicity and T cell receptor signaling) aligns with the central role of NK/T-cell–mediated tumor surveillance and effector function within the LUAD tumor microenvironment [52]. Checkpoint and cytokine signaling (PD-L1 expression and PD-1 checkpoint pathway in cancer together with Interferon gamma signaling) aligns with prior evidence that IFN-γ–driven activation programs can also promote adaptive immune resistance via PD-L1 upregulation [53–55]. Finally, enrichment of Signaling by Receptor Tyrosine Kinases and the VEGFA–VEGFR2 Pathway is compatible with canonical growth and angiogenesis programs in NSCLC, which are also known to interact with immune regulation in the tumor microenvironment [56–58].

Across diseases, the importance score distributions highlight biologically coherent modules, suggesting that the attention-derived, PPI-constrained geometry together with feature-scaled importance scores helps capture disease-associated signaling patterns that are compact, connected, and functionally interpretable.

## 3 Dicussion

Tissue-level and single-cell omic resources are being generated at unprecedented scale to interrogate disease pathogenesis—the core of precision medicine. Graph neural networks (GNNs) have been widely used to integrate molecular measurements with interaction knowledge for target identification and pathway inference [34, 59–61]. Nevertheless, despite strong predictive performance, prevailing graph-based approaches that operate on numeric, expression-centric signaling graphs capture only part of the scientific discovery workflow: they often underutilize the rich, human-interpretable priors encoded in biological text and curated knowledge bases. To address this gap, this work introduces a three-part ecosystem that unifies data, tooling, and modeling. First, **OmniCellTOSG** is a large-scale single-cell text–omic signaling graph resource whose **Text–Omic Signaling Graphs (TOSGs)** couple text-attributed biological knowledge with numerical gene/protein abundance, enabling graph-structured decoding of cellular signaling across tissues, diseases, ages, and conditions. Second, CellTOSG_Loader provides a NumPy-ready query–load–balance pipeline that constructs stratified, unbiased cohorts across user-specified facets (e.g., cell type, tissue, disease, data source, age/sex), mitigates class imbalance, and applies batch correction, standardizing ingestion and ensuring fair, repeatable experimentation. Third, the **CellTOSG Foundation Model (CellTOSG-FM)** integrates a graph-language architecture that jointly encodes biological textual priors, quantitative omic measurements, and signaling topology over TOSGs. By enabling message passing on knowledge-grounded graphs while aligning cross-modal representations, the model learns structure-aware embeddings that support downstream tasks including cell-type annotation, cell-attribute classification, and signaling inference with interpretable subgraph rationales.

These properties position TOSGs as a natural substrate for foundation-model training, enabling the learning of broadly transferable model of cellular signaling. Pretraining **CellTOSG-FM** on massive, heterogeneous TOSG corpora from **OmniCellTOSG** via self-supervised learning, which leverages edge-masked reconstruction that emphasizes signaling network structure, yields broadly transferable model of signaling patterns and provides robust bases for task-specific adaptation, outperforming disease- or dataset-specific pipelines that risk bias and overfitting. The **OmniCellTOSG** dataset is openly available in a PyTorch-friendly format, lowering barriers to reproducible benchmarking and catalyzing community development of graph language foundation models for precision medicine over cellular systems. Together, **OmniCellTOSG**, CellTOSG_Loader, and **CellTOSG-FM** establishes a scalable, mechanism-oriented framework for cell type annotation, disease classification, subtype delineation, and targets and signaling networks graph inference. Ongoing curation continues to expand its coverage across diseases, tissues, sex, age, and diverse experimental conditions, enabling improved interrogation of complex signaling programs and the prioritization of actionable perturbations, including candidate drugs and rational combinations that target dysfunctional nodes and pathways.

## 4 Methods

### 4.1 Data Collection and Preprocessing

The dataset was compiled primarily from three large sources, with additional cohorts added to broaden tissue and disease coverage (collection procedures in Section 5.1). From CellxGene, we obtained over 71 million single cells/nuclei across 65 human tissues and 125 disease studies in H5AD AnnData format [1, 2]; from the Brain Cell Atlas, over 7 million human brain single cells spanning 21 disease types [4, 62]; and from GEO, four studies contributing over 650,000 cells to fill underrepresented indications. We further integrated the Hepatitis Atlas to include hepatitis C virus infection data (over 7,000 cells) and the Human Cell Atlas to expand pancreas coverage with over 98,000 cells and three additional disease conditions. All datasets were converted to a unified H5AD schema to support a standardized preprocessing workflow. The resulting preprocessed resource comprises over 79 million high-quality cells covering 762 cell types, with samples organized and split by source, coarse- and fine-grained tissue labels, disease, and suspension type.

To mitigate the inherent sparsity and noise in sc/snRNA-seq data, we adopt a meta-cell strategy based on the SEACells algorithm[19]. Our approach is designed to ensure consistency across datasets from diverse sources by employing uniform preprocessing, feature selection, and dimensionality reduction procedures before meta-cell aggregation. Let the raw data be represented by ${𝒳}^{\left(\alpha \right)}=\left\{X_{1}^{\left(\alpha \right)},X_{2}^{\left(\alpha \right)},\cdots ,X_{n_{0}}^{\left(\alpha \right)},\cdots ,X_{N_{0}}^{\left(\alpha \right)}\right\}$, where $X_{n_{0}}^{\left(\alpha \right)}\in R^{M_{0}}$ denotes the cell, and $N_{0}$ is the number of cells collected from various data resources and $M_{0}$ is the number of elements in transcript entity set $𝒯=\left\{T_{1},T_{2},\cdots ,T_{m_{0}},\cdots ,T_{M_{0}}\right\}$. For computational demands, raw data files (stored in H5AD format) are partitioned into subsets of no more than 50,000 cells. For datasets requiring normalization, we first apply total count normalization by scaling UMI counts of each cell to a fixed total of 10,000, followed by a log1p transformation to stabilize variance. In addition, Uniform feature selection is performed by identifying the top 1,500 highly variable genes from each dataset. We then apply Principal Component Analysis (PCA[63]) with 50 components to reduce dimensionality while preserving essential variance. Based on the PCA-reduced features, a K-Nearest Neighbor (KNN[64]) graph is constructed to maintain the underlying structural relationships among cells. Meta-cell generation is performed using the SEACells algorithm. With a fixed aggregation size of N cells per meta cell, SEACells first measures cell-to-cell similarity and then decomposes the resulting structure via archetypal analysis. Cells near the convex hulls of the data distribution are grouped together, yielding a new set of meta cells denoted by ${𝒳}^{\left(\beta \right)}=\left\{X_{1}^{\left(\beta \right)},X_{2}^{\left(\beta \right)},\cdots ,X_{n}^{\left(\beta \right)},\cdots ,X_{N}^{\left(\beta \right)}\right\}$, where $X_{n}^{\left(\beta \right)}\in R^{M_{0}}$ represents a meta-cell.

Correspondingly, the associated attributes (e.g., sex, cell_type, development_stage, tissue, disease_status, etc.) for the meta cells are computed by aggregating the raw cell attributes through majority voting, resulting with ${𝒜}^{\left(\beta \right)}$ (see Figure 1a). After all meta-cells are constructed, we group the data by source and tissue (coarse-grained), read the corresponding meta-cell H5AD files, and map gene identifiers to the BioMedGraphica framework (see Section 4.2). The expanded expression matrices are then serialized into NumPy shards of 10,000 samples per file. For each sample, we record the relative path of the NumPy matrix (matrix_file_path) and the corresponding row index in the matrix file (matrix_row_idx); these pointers are stored together with the H5AD .obs fields and saved in CSV format for downstream processing. Because the datasets originate from diverse sources, the nomenclature of attributes such as cell type, disease, development stage, and sex varies substantially. To standardize cell type, we built a mapping pipeline using the unique cell type values extracted from all datasets and a cell type mapping table (CMT) based on Cell Ontology (CL) database. All synonym fields in the cell type mapping table were expanded to generate a candidate dictionary linking each synonym to its CMT ID and the corresponding CL label. Before matching, anchor rules were defined to handle specific terms and generic placeholders such as “unknown” or “unclassified” were ignored. For every original cell type, exact matching was first attempted; if not found, fuzzy matching (token-sort ratio) was applied to compute the best-scoring candidate. The resulting pairs contained the original term, the matched CMT term, CMT ID, CL label, and the matching score. Terms with scores lower than 100 were manually reviewed and corrected, producing a curated mapping that unified all cell type under the Cell Ontology standard. The same procedure was used for disease terms to obtain BMG disease identifiers.

The development stage values were normalized by converting free-text descriptions into approximate numeric ages (in years) using regular-expression parsing of units such as years, months, weeks, days, and Carnegie stages, followed by categorization into MeSH-based age groups (e.g., infant, child, young adult, middle aged)[65]. Each entry was also assigned a coarse birth phase label (pre-birth, post-birth, or unknown). For sex, all terms were normalized through direct mapping (e.g., f for female, m for male), with unrecognized or empty values set to unknown. Finally, by integrating the metadata with the curated mapping results, we obtain the standardized attribute set $𝒜={\left\{a_{i}\right\}}_{i=1}^{m}$ (Table S2). This set serves as retrieval metadata for CellTOSG_Loader to locate and extract the corresponding cells (Section 4.3).

### 4.2 OmniCellTOSG Generation

With the preprocessed single-cell transcriptomic dataset denoted as ${𝒳}^{\left(\beta \right)}\in R^{N\times M_{0}}$, we integrate it into the BioMedGraphica framework together with the gene-regulatory network. Using the mapping table, the $M_{0}$ transcript features are mapped to $M_{t}$ transcript entities. Specifically, each transcript element in the set 𝒯 is mapped and extended to the transcript-entity set ${𝒱}^{\left(t\right)}=\left\{v_{1}^{\left(t\right)},v_{2}^{\left(t\right)},\ldots ,v_{M_{t}}^{\left(t\right)}\right\}$. By linking transcript nodes within the network to the protein–protein interaction (PPI) graph, proteins are treated as virtual nodes, yielding the additional entity set ${𝒱}^{\left(p\right)}$. The overall entity set is $𝒱=\left\{{𝒱}^{\left(t\right)},{𝒱}^{\left(p\right)}\right\}$, with $|𝒱|=\left|{𝒱}^{\left(t\right)}\right|+\left|{𝒱}^{\left(p\right)}\right|=M_{t}+M_{p}=M$. Likewise, the feature set $𝒳=\left\{{𝒳}^{\left(t\right)},{𝒳}^{\left(p\right)}\right\}$ is generated, where $𝒳\in R^{N\times M},{𝒳}^{\left(t\right)}\in R^{N\times M_{t}}$, and ${𝒳}^{\left(p\right)}\in R^{N\times M_{p}}$ correspond to the transcriptomic and proteomic feature sets, respectively.

From the perspective of single cell side, the multi-omics 𝒳 can be decomposed as $\left\{X_{1},X_{2},\cdots ,X_{n},\cdots ,X_{N}\right\}$, where each sample $X_{n}$ resides in $R^{M}$. Additionally, the cell label matrices set 𝒴, and given that the cell label set are consistent with label for meta cells, ${𝒴}^{\left(\beta \right)}$. Beyond transcriptomic features and virtual proteomic features, an auxiliary node textual information dataset, $𝒮=\left\{S^{\left(\varphi \right)},S^{\left(\chi \right)},S^{\left(\psi \right)}\right\}$, is incorporated. Each of those entity textual information correpsonds to the node in entity set 𝒱. The $S^{\left(\varphi \right)}=\left[s_{1}^{\left(\varphi \right)},s_{2}^{\left(\varphi \right)},\cdots ,s_{m}^{\left(\varphi \right)},\cdots ,s_{M}^{\left(\varphi \right)}\right]$, representing the entity names (e.g., HGNC symbol, Ensembl ID), $S^{\left(\chi \right)}=\left[s_{1}^{\left(\chi \right)},s_{2}^{\left(\chi \right)},\cdots ,s_{m}^{\left(\chi \right)},\cdots ,s_{M}^{\left(\chi \right)}\right]$, representing the entity textual descriptions (e.g., Uniprot protein description), and $S^{\left(\psi \right)}=\left[s_{1}^{\left(\psi \right)},s_{2}^{\left(\psi \right)},\cdots ,s_{m}^{\left(\psi \right)},\cdots ,s_{M}^{\left(\psi \right)}\right]$, representing biochemical information (i.e., RNA sequences or protein sequences). Therefore, for any entity, $v_{m}$, it has the textual information set $s_{m}=\left\{s_{m}^{\left(\varphi \right)},s_{m}^{\left(\chi \right)},s_{m}^{\left(\psi \right)}\right\}$. And the entity textual information dataset, 𝒮, enhances the graph’s expressivity, facilitating the generation of a textual-attributed transcriptomic signaling knowledge graph.

Afterwards, to construct the text–omic signaling graph 𝒢=(𝒱,ℰ), we identify relations (edges) between entities. As noted above, the vertex set is $𝒱=\left\{{𝒱}^{\left(t\right)},{𝒱}^{\left(p\right)}\right\}$. We consider two relation types: internal signaling and PPI-based gene-regulatory signaling. Accordingly, the graph decomposes into the internal-signaling subgraph ${𝒢}^{\text{(in)}}=\left({𝒱}^{\text{(in)}},{ℰ}^{\text{(in)}}\right)$, which captures the molecular flow from transcripts to proteins, and the PPI-regulatory subgraph ${𝒢}^{\left(\text{PPI}\right)}=\left({𝒱}^{\left(\text{PPI}\right)},{ℰ}^{\left(\text{PPI}\right)}\right)$, which captures protein–protein interactions, with the overall edge set $ℰ={ℰ}^{\text{(in)}}\cup {ℰ}^{\text{(PPI)}}$. By construction, ${𝒱}^{\left(\text{in}\right)}=𝒱$ with $\left|{𝒱}^{\left(\text{in}\right)}\right|=M=M_{t}+M_{p}$, while ${𝒱}^{\left(\text{PPI}\right)}={𝒱}^{\left(p\right)}$.

Overall, the pipeline condenses 79,195,364 raw cells into N=395,317 meta-cells and aligns M=533,458 molecular entities (transcript/protein nodes) enriched with textual and topological information with internal signaling edges with $\left|{ℰ}^{\left(\text{in}\right)}\right|=152,585$ and PPI-regulatory subgraph with $\left|{ℰ}^{\left(\text{PPI}\right)}\right|=16,484,820$. Building on these components, we fuse preprocessed single-cell transcriptomic profiles with prior gene–regulatory and signaling knowledge to construct TOSGs, and we release the dataset 𝒟=(𝒳,𝒜,𝒮,ℰ). The resulting TOSGs provide a unified, graph-structured substrate for foundation-model pretraining and downstream tasks by coupling numeric omics measurements with textual and topological knowledge, thereby enabling structure-aware learning and interpretable signaling analysis. A comprehensive summary of organ and disease coverage is provided in Table S3.

### 4.3 CellTOSG_Loader Package

To enable scalable access, the feature matrix $𝒳\in R^{N\times M}$ in **OmniCellTOSG** is partitioned row-wise into fixed-size NumPy shards, each stored as an x.npy file with lightweight metadata recording global row indices. After downloading the dataset to a local root, users employ CellTOSG_Loader (Appendix 5.3.1), which discovers the relevant shards and materializes only the requested subset, thereby avoiding full-matrix loads. Cohorts are defined via standardized metadata filters conditions (e.g., {tissue_general: brain, disease_name: Alzheimer’s Disease}) expressed over the attribute set 𝒜; the supervised objective and target field are designated by task and label_column, respectively. Given these inputs, the loader deterministically translates user arguments into a formal query, extracts the feasible subset from 𝒳, and optionally applies subsampling (to optimize memory usage), class balancing, and batch correction via ComBat–seq [25, 26]. At the core of retrieval is a two-phase Stratified Retrieval Algorithm (SRA) tailored to 𝒳 and its named attributes 𝒜 as meta data. In **Phase I (query-constrained extraction)**, a user’s conjunctive query yields the subset R(q) by enforcing that all specified attribute constraints hold. In **Phase II (task-aware balancing)**, a task configuration specifies the balance label and control value, the exact-match covariates, and an ordered age–stage key; cases are taken from R(q) (non-control label), controls are drawn by reapplying the same filters with the label fixed to the control value, and key-stratified matching is performed: non-age covariates must match exactly within each stratum, while age differences are bounded by a tolerance δ along the ordered age–stage axis (with optional upsampling and discarding infeasible strata). The outcome is a stratified cohort ${𝒳}_{R}$ whose non-stage covariates are balanced by construction and whose age-stage offsets satisfy $d_{k^{⋆}}\leq \delta$. For cell-type annotation, balancing is disabled and rare types (<μ samples) may be upsampled for training stability. The procedure simultaneously returns the label set $Y_{R}$ aligned with ${𝒳}_{R}$. The Stratified Retrieval Algorithm is described in detail below.

In the Query-Constrained Extraction phase, we let 𝒳 be the set of samples and 𝒜 the set of attributes. For each a∈𝒜 with value space $\Sigma_{a}$, define the attribute–evaluation map $u_{a}:𝒳\to \Sigma_{a},x\mapsto u_{a}(x)$. Equivalently, collect these into a single evaluation map $u:𝒳\times 𝒜\to {⋃}_{a\in 𝒜}\Sigma_{a}$ via $u(x,a):=u_{a}(x)$. A user query is a finite set of attribute constraints $q={\left\{\left(a,V_{a}\right)\right\}}_{a\in \text{dom}(q)}$ with nonempty admissible sets $V_{a}\subseteq \Sigma_{a}$ and $\text{dom}(q)=\left\{a\in 𝒜:V_{a}\neq \emptyset \right\}$, interpreted by the conjunctive predicate

(1) $$Q_{q}\left(x\right)=\underset{\left(a,V_{a}\right)\in q}{⋀}ℐ\left\{u_{a}\left(x\right)\in V_{a}\right\},$$

where ℐ{⋅}∈{0,1} equals 1 when the statement is true and 0 otherwise. The selection induced by the query is the feasible set

(2) $$R(q)≔\left\{x\in 𝒳:Q_{q}(x)=1\right\}=\underset{\left(a,V_{a}\right)\in q}{⋂}u_{a}^{-1}\left(V_{a}\right),$$

i.e., the set of samples that satisfy all attribute-wise constraints simultaneously.

During Phase II of Task-Aware Balancing, we Let the task configuration be $\Lambda (\lambda )=\left(b,b_{0},K,k^{⋆}\right)$, where b∈𝒜 is the balance field with control value $b_{0}\in \Sigma_{b}$, and $K=\left(k_{1},\ldots ,k_{r}\right)\subseteq 𝒜$ is the ordered tuple of match keys with designated age–stage key $k^{⋆}=k_{j^{⋆}}$ for some index $1\leq j^{⋆}\leq r$. Define the query with any constraint on b removed by

(3) $$q_{-b}=\left\{\left(a,V_{a}\right)\in q:a\neq b\right\}.$$

The control pool applies the same non-b filters and overwrites b to its control value:

(4) $$\text{NM}\left(\lambda ,q\right)=\left\{x\in 𝒳:u_{b}\left(x\right)=b_{0}\wedge Q_{q_{-b}}\left(x\right)=1\right\}.$$

The case set is drawn from the query subset:

(5) $$\text{CA}=\left\{x\in R(q):u_{b}(x)\neq b_{0}\right\}.$$

Let $\Sigma_{K}:=\Sigma_{k_{1}}\times \cdots \times \Sigma_{k_{r}}$ and define the key map

(6) $$\kappa_{K}:𝒳\to \Sigma_{K},\;\kappa_{K}(x)=\left(u_{k_{1}}(x),\ldots ,u_{k_{r}}(x)\right).$$

For any key tuple $\kappa \in \Sigma_{K}$, define the strata

(7) $${\text{CA}}_{\kappa }=\left\{x\in \text{CA}:\kappa_{K}(x)=\kappa \right\},\;{\text{NM}}_{\kappa }=\left\{x\in \text{NM}(\lambda ,q):\kappa_{K}(x)=\kappa \right\}.$$

Endow $\left(\Sigma_{k^{⋆}},⪯\right)$ with a rank map $\rho :\Sigma_{k^{⋆}}\to \{0,1,\ldots ,L\}$ and induced distance

(8) $$d_{k^{⋆}}\left(e,e^{\prime }\right):=\left|\rho (e)-\rho \left(e^{\prime }\right)\right|.$$

Given a tolerance δ≥0, the offset-admissible control pool for stratum κ is

(9) $${𝒩}_{\kappa }^{\left(\delta \right)}=\left\{x\in \text{NM}(\lambda ,q):u_{k_{i}}(x)=\kappa_{i}\forall i\neq j^{⋆},d_{k^{⋆}}\left(u_{k^{⋆}}(x),\kappa_{j^{⋆}}\right)\leq \delta \right\}.$$

Matching proceeds per stratum by first taking exact-stage controls $\left(d_{k^{⋆}}=0\right)$; if insufficient, progressively admitting offsets 1,2,…,δ according to the stage order, sampling without replacement within each offset layer, and finally (if enabled) upsampling with replacement from the collected controls to meet the stratum size. Strata with no admissible controls are discarded. Let ${\text{CA}}_{\kappa }^{\prime }$ and ${\text{NM}}_{\kappa }^{\prime }$ denote the retained case rows and matched controls for successful strata. The balanced output is

(10) $${𝒳}_{R}=\underset{\kappa \in {𝒦}^{*}}{⋃}\left({\text{CA}}_{\kappa }^{\prime }\cup {\text{NM}}_{\kappa }^{\prime }\right),\;\text{with}\;\left|{\text{CA}}_{\kappa }^{\prime }\right|=\left|{\text{NM}}_{\kappa }^{\prime }\right|\forall \kappa \in {𝒦}^{*},$$

where ${𝒦}^{*}$ is the set of strata that achieved feasible matching. Within each retained stratum, all non-stage keys in K match exactly by construction, and stage differences satisfy $d_{k^{⋆}}\leq \delta$. Finally, the task-specific labels $Y_{R}$ are obtained by restricting the label map $\ell_{b}:𝒳\to \Sigma_{b}$ to the returned cohort, i.e., $Y_{R}=\left\{\ell_{b}(x):x\in {𝒳}_{R}\right\}$, which is one-to-one aligned with the rows. The details of the algorithm can be checked in the Appendix 5.3.2.

### 4.4 Graph Language Foundation Model

#### 4.4.1 CellTOSG-FM pretraining

Given the integrated text–omic signaling graph dataset 𝒟, which comprises a single-cell text–omic signaling graph 𝒢=(𝒱,ℰ) together with text–omic feature sets 𝒳 and 𝒮, we construct a self-supervised pretraining task by sampling a subset ${𝒳}_{\text{pre}}\subseteq 𝒳$. For edge-masking pretraining, we draw an edge mask set ${ℰ}_{\text{mask}}\sim \text{Bernoulli}(p)$ over the protein–protein interaction subset ${ℰ}^{\left(\text{PPI}\right)}$, where p∈(0,1) denotes the masking ratio used to occlude signaling flow along PPI edges. The foundation model is then pretrained via

(11) $$ℋ=f_{\text{pre}}\left({𝒳}_{\text{pre}},𝒮,ℰ,{ℰ}_{\text{mask}}\right)$$,

where $ℋ\in R^{N\times M\times d}$ is the entity embeddings, and $f_{\text{pre}}(\cdot )$ is the pre-trained foundation model. In details, to merge the text-omics feature sets 𝒳,𝒮 into unified entity embeddings, bi-encoder framework was leveraged by

(12) $${𝒳}^{\prime }={\text{ENC}}_{\text{Omic}}^{\text{pre}}\left({𝒳}_{\text{pre}}\right)$$

(13) $${𝒮}^{\prime }={\text{ENC}}_{\text{Text}}(𝒮)$$

(14) $${ℋ}^{\prime }={\text{ENC}}_{\text{Cross}}^{\text{pre}}\left({𝒳}^{\prime },{𝒮}^{\prime }\right)$$,

where the ${\text{ENC}}_{\text{Omic}}^{\text{pre}}(\cdot )$ is the linear transformation and ${𝒳}^{\prime }\in R^{N\times M\times d^{\prime }}$ the ${\text{ENC}}_{\text{Text}}(\cdot )$ can be BERT-based or other language models (LMs) and ${𝒮}^{\prime }=\left\{S^{\left(\gamma \right)},S^{\left(\theta \right)},S^{\left(\rho \right)}\right\}$, where $S^{\left(\gamma \right)}\in R^{M\times d^{\prime }},S^{\left(\theta \right)}\in R^{M\times d^{\prime }},S^{\left(\rho \right)}\in R^{M\times d^{\prime }}$ are encoded as entity name, textual description and biochemical embeddings. The ${\text{ENC}}_{\text{Cross}}^{\text{pre}}(\cdot )$ will fuse the omic embeddings and the textual embeddings to generate ${ℋ}^{\prime }\in R^{N\times M\times d^{\prime }}$.

Afterwards, the internal signaling will be propagated by using graph encoder with

(15) $${ℋ}^{\left(\text{in}\right)}={\text{GNN}}_{\text{in}}^{\text{pre}}\left({ℋ}^{\prime },{ℰ}^{\left(\text{in}\right)}\right)$$,

where ${\text{GNN}}_{\text{in}}^{\text{pre}}(\cdot )$ uses message propagation via GNN layers and ${ℋ}^{\left(\text{in}\right)}\in R^{N\times M\times d}$. Finally, with the prepared entity embedding, the foundation model will be pretrained by masking nodes with

(16) $$ℋ={\text{GNN}}_{\text{global}}^{\text{pre}}\left({\text{MLP}}_{\text{pre}}\left({ℋ}^{\left(\text{in}\right)}\right),{ℰ}^{\left(\text{PPI}\right)},{ℰ}_{\text{mask}}\right)$$,

where ${\text{GNN}}_{\text{global}}^{\text{pre}}(\cdot )$ denotes global signaling message propagation via GNN layers and ${\text{MLP}}_{\text{pre}}(\cdot )$ represents the multilayer perceptron to embed the node features before the final stage of pretraining.

To update the pretraining model parameters, we adopt a reconstruction objective. Specifically, we define the set of visible edges as ${ℰ}_{\text{vis}}={ℰ}^{\left(\text{PPI}\right)}\setminus {ℰ}_{\text{mask}}$. The structural decoder $u_{\omega }$ with parameters ω outputs the probability of an edge between nodes $m_{i}$ and $m_{j}$ as

(17) $$u_{\omega }\left(h_{m_{i}}^{\left(n\right)},h_{m_{j}}^{\left(n\right)}\right)=\sigma \left({\text{MLP}}_{\omega }\left(h_{m_{i}}^{\left(n\right)}⊙h_{m_{j}}^{\left(n\right)}\right)\right),$$

where $h_{m_{i}}^{\left(n\right)},h_{m_{j}}^{\left(n\right)}\in R^{d}$ are the global node embeddings for nodes $m_{i}$ and $m_{j}$ in sample n; MLP denotes a multilayer perceptron and ⊙ the element-wise product. In parallel, the degree decoder $v_{\phi }$ with parameters ϕ predicts the node degree via

(18) $$v_{\phi }\left(h_{m}^{\left(n\right)}\right)={\text{MLP}}_{\phi }\left(h_{m}^{\left(n\right)}\right)$$

We compute the pretraining reconstruction loss as the sum of an edge-reconstruction term and a degree-reconstruction term, with ${ℰ}^{+}={ℰ}_{\text{vis}}$ and a set of sampled negative edges ${ℰ}^{-}$ drawn from pairs not in ${ℰ}^{\text{PPI}}$. The binary cross-entropy edge losses for sample n are

(19) $${ℒ}_{+}^{\left(n\right)}=\frac{1}{\left|{ℰ}^{+}\right|}\sum_{\left(m_{i},m_{j}\right)\in {ℰ}^{+}}\text{log}\;u_{\omega }\left(h_{m_{i}}^{\left(n\right)},h_{m_{j}}^{\left(n\right)}\right)$$

(20) $${ℒ}_{-}^{\left(n\right)}=\frac{1}{\left|{ℰ}^{-}\right|}\sum_{\left(m_{i},m_{j}\right)\in {ℰ}^{-}}\text{log}\left(1-u_{\omega }\left(h_{m_{i}}^{\left(n\right)},h_{m_{j}}^{\left(n\right)}\right)\right)$$

(21) $${ℒ}_{\text{edge}}^{\left(n\right)}=-\left({ℒ}_{+}^{\left(n\right)}+{ℒ}_{-}^{\left(n\right)}\right)$$

and the degree-reconstruction loss is

(22) $${ℒ}_{\text{deg}}^{\left(n\right)}=\frac{1}{\left|𝒱\right|}\sum_{p\in 𝒱}{\left\|v_{\phi }\left(h_{m}^{\left(n\right)}\right)-\text{deg}\left(m^{\left(n\right)}\right)\right\|}^{2}$$

where deg(⋅) denotes the degree of node m for sample cell n in the graph ${𝒢}^{\left(\text{PPI}\right)}$.

With optional weights $\lambda_{\text{edge}},\lambda_{\text{deg}}>0$, the per-sample objective and the dataset-averaged pretraining loss are

(23) $${ℒ}^{\left(n\right)}=\lambda_{\text{edge}}{ℒ}_{\text{edge}}^{\left(n\right)}+\lambda_{\text{deg}}{ℒ}_{\text{deg}}^{\left(n\right)},\;{ℒ}_{\text{pre}}=\frac{1}{N}\sum_{n=1}^{N}{ℒ}^{\left(n\right)}.$$

This objective jointly optimizes the encoder and both decoders to reconstruct the masked PPI topology and node degrees from ℋ, thereby aligning the learned embeddings with the global signaling structure.

### 4.5 Model Downstream Tasks

Ultimately, the objective is to use the pretrained foundation model, $f_{\text{pre}}(\cdot )$, that synergistically integrates the task specific incoming feature set ${𝒳}_{\tau }\in R^{N_{\tau }\times M}$, node descriptions 𝒮, and graph topology ℰ to predict cell-specific outcomes. As to the unsupervised task, the latent embedding for the incoming feature set will be generated by

(24) $${ℋ}^{\left(\tau \right)}=f_{\text{pre}}\left({𝒳}_{\tau },𝒮,ℰ\right)$$,

where ${ℋ}^{\left(\tau \right)}\in R^{N_{\tau }\times M\times d}$. For supervised learning, the foundation model will predict the cell outcomes by

(25) $${\overset{ˆ}{𝒴}}_{\tau }=f_{\text{down}}\left({ℋ}^{\left(\tau \right)},ℰ,{𝒮}^{\prime }\right)$$,

where ${\overset{ˆ}{𝒴}}_{\tau }\in R^{N}$ represents the predicted cellular states, which depends on specific downstream tasks (e.g., cell type annotations or celluar condition (normal vs. disease)) and $f_{\text{down}}(\cdot )$ is the downstream decoder, which contains following components of encoders,

(26) $${ℋ}_{\text{Cross}}^{\left(\tau \right)}={\text{ENC}}_{\text{Cross}}^{\text{down}}\left({\text{ENC}}_{\text{Omic}}^{\text{down}}\left({ℋ}^{\left(\tau \right)}\right),{𝒮}^{\prime }\right)$$

(27) $${𝒵}^{\left(\tau \right)}={\text{GNN}}_{\text{global}}^{\text{down}}\left({\text{GNN}}_{\text{in}}^{\text{down}}\left({ℋ}_{\text{Cross}}^{\left(\tau \right)},ℰ\right)\right)$$

(28) $${\overset{ˆ}{𝒴}}_{\tau }={\text{MLP}}_{\text{down}}\left({𝒵}^{\left(\tau \right)}\right)$$,

where ${\text{ENC}}_{\text{Omic}}^{\text{down}}(\cdot )$ and ${\text{ENC}}_{\text{Cross}}^{\text{down}}(\cdot )$ represent the downstream omic encoder and downstream cross-modality encoder, ${\text{GNN}}_{\text{in}}^{\text{down}}(\cdot )$ and ${\text{GNN}}_{\text{global}}^{\text{down}}(\cdot )$ are downstream internal signaling and global signaling message propagation via GNN layers and ${\text{MLP}}_{\text{down}}(\cdot )$ is the linear classifier. To infer the cell-specific targets and signaling networks network for sample $n_{\tau }$, an affinity matrix for measuring the attention-based edge weight will be derived from ${𝒵}_{n_{\tau }}^{\left(\tau \right)}$, the latent space embeddings of sample $n_{\tau }$, by

(29) $$A_{n_{\tau }}^{\left(\tau \right)}=\text{ATT}\left({𝒵}_{n_{\tau }}^{\left(\tau \right)}\right)$$,

where ATT(⋅) is an attention-based similarity function. To restrict the edge weights to the protein–protein interaction (PPI) topology, we use

(30) $${\left(W_{ℰ}^{\left(\tau \right)}\right)}_{n_{\tau }}=\text{TRAN}\left(A_{n_{\tau }}^{\left(\tau \right)}\right)⊙\text{ONEHOT}\left({ℰ}^{\left(\text{PPI}\right)}\right)$$,

where TRAN(⋅) is the transformation function to trun the adjacency matrix to edge pairs in dimensions of |ℰ| by 2 and ${\left(W_{ℰ}^{\left(\tau \right)}\right)}_{n_{\tau }}\in R^{\left|{ℰ}_{\text{PPI}}\right|\times 2}$ are cell specific PPI edge weights for sample $n_{\tau }$. To calculate the node importance score for certain node m in the sample $n_{\tau }$, we integrate the averaged bi-directional edge weights and omic values by

(31) $${\left(W_{𝒱}^{\left(\tau \right)}\right)}_{n_{\tau }}^{\left(m\right)}=\sum_{i}\left[{\left(W_{ℰ}^{\left(\tau \right)}\right)}_{mi}+{\left(W_{ℰ}^{\left(\tau \right)}\right)}_{im}\right]$$,

where ${\left(W_{𝒱}^{\left(\tau \right)}\right)}_{n_{\tau }}^{\left(m\right)}$ are node importance score for node m in sample $n_{\tau }$. Finally, a core-extraction routine will select a compact subgraph by

(32) $${𝒢}_{n_{\tau }}^{\left(\tau \right)}=f_{\text{core}}\left({\left(W_{𝒱}^{\left(\tau \right)}\right)}_{n_{\tau }},{\left(W_{ℰ}^{\left(\tau \right)}\right)}_{n_{\tau }};\xi ,\epsilon \right),$$,

where the operator $f_{\text{core}}(\cdot )$ ranks nodes by ${\left(W_{𝒱}^{\left(\tau \right)}\right)}_{n_{\tau }}$ to retain the top ξ nodes and applies a branch-pruning procedure [35] to keep the top ϵ edges in ${\left(W_{ℰ}^{\left(\tau \right)}\right)}_{n_{\tau }}$, yielding the core subgraph ${𝒢}_{n_{\tau }}^{\left(\tau \right)}=\left({𝒱}_{n_{\tau }}^{\left(\tau \right)},{ℰ}_{n_{\tau }}^{\left(\tau \right)}\right)$.

## Supplementary Material

1

## Data Availability

The OmniCellTOSG dataset is available on Hugging Face: https://huggingface.co/datasets/FuhaiLiAiLab/OmniCellTOSG_Dataset. All dataset sources used in this study are documented in Data Collection (Section 5.1). Detailed information for datasets collected from GEO and other repositories is provided in Table S1. Datasets from CELLxGENE were retrieved through the CELLxGENE Census (release version 2025–01-30) and the CELLxGENE portal (https://chanzuckerberg.github.io/cellxgene-census/python-api.html, https://cellxgene.cziscience.com/). The preprocessed GSE129308 dataset used in downstream evaluations and other Brain Cell Atlas data used in dataset construction were downloaded from: https://www.braincellatlas.org/dataSet.

## Code availability:

The CellTOSG_Loader package and the CellTOSG-FM foundation model code are available on GitHub: https://github.com/FuhaiLiAiLab/OmniCellTOSG

## References

[1] Megill C., Martin B., Weaver C., Bell S., Prins L., Badajoz S., McCandless B., Pisco A.O., Kinsella M., Griffin F., : Cellxgene: a performant, scalable exploration platform for high dimensional sparse matrices. BioRxiv, 2021–04 (2021)

[2] Program C.C.S., Abdulla S., Aevermann B., Assis P., Badajoz S., Bell S.M., Bezzi E., Cakir B., Chaffer J., Chambers S., : Cz cellxgene discover: a single-cell data platform for scalable exploration, analysis and modeling of aggregated data. Nucleic Acids Research 53(D1), 886–900 (2025) 10.1093/nar/gkae1142 PMC11701654 39607691

[3] Rood J.E., Wynne S., Robson L., Hupalowska A., Randell J., Teichmann S.A., Regev A.: The human cell atlas from a cell census to a unified foundation model. Nature, 1–2 (2024) 10.1038/s41586-024-08338-4 39566552

[4] Chen X., Huang Y., Huang L., Huang Z., Hao Z.-Z., Xu L., Xu N., Li Z., Mou Y., Ye M., : A brain cell atlas integrating single-cell transcriptomes across human brain regions. Nature Medicine 30(9), 2679–2691 (2024) 10.1038/s41591-024-03150-z PMC11405287 39095595

[5] Miller J.A., Hawrylycz M.J., Aitken M., Ariza J., Chakrabarty R., Ding S.-L., Ding Y., Ferrer R., Goldy J., Gratiy S., : Sea-ad: Scientific analysis and open access resources targeting early changes in alzheimer’s disease. Alzheimer’s & Dementia 19, 063478 (2023)

[6] Mathys H., Peng Z., Boix C.A., Victor M.B., Leary N., Babu S., Abdelhady G., Jiang X., Ng A.P., Ghafari K., : Single-cell atlas reveals correlates of high cognitive function, dementia, and resilience to alzheimer’s disease pathology. Cell 186(20), 4365–4385 (2023) 37774677 10.1016/j.cell.2023.08.039 PMC10601493

[7] Heimberg G., Kuo T., DePianto D.J., Salem O., Heigl T., Diamant N., Scalia G., Biancalani T., Turley S.J., Rock J.R., : A cell atlas foundation model for scalable search of similar human cells. Nature, 1–3 (2024) 10.1038/s41586-024-08411-y PMC11864978 39566551

[8] Theodoris C.V., Xiao L., Chopra A., Chaffin M.D., Al Sayed Z.R., Hill M.C., Mantineo H., Brydon E.M., Zeng Z., Liu X.S., : Transfer learning enables predictions in network biology. Nature 618(7965), 616–624 (2023) 37258680 10.1038/s41586-023-06139-9 PMC10949956

[9] Cui H., Wang C., Maan H., Pang K., Luo F., Duan N., Wang B.: scgpt: toward building a foundation model for single-cell multi-omics using generative ai. Nature Methods 21(8), 1470–1480 (2024) 38409223 10.1038/s41592-024-02201-0

[10] Hao M., Gong J., Zeng X., Liu C., Guo Y., Cheng X., Wang T., Ma J., Zhang X., Song L.: Large-scale foundation model on single-cell transcriptomics. Nature methods 21(8), 1481–1491 (2024) 38844628 10.1038/s41592-024-02305-7

[11] Yuan X., Zhan Z., Zhang Z., Zhou M., Zhao J., Han B., Li Y., Tang J.: Cell ontology guided transcriptome foundation model. Advances in Neural Information Processing Systems 37, 6323–6366 (2024)

[12] Hou Z., Liu X., Cen Y., Dong Y., Yang H., Wang C., Tang J.: Graphmae: Self-supervised masked graph autoencoders. In: Proceedings of the 28th ACM SIGKDD Conference on Knowledge Discovery and Data Mining, pp. 594–604 (2022)

[13] Li J., Wu R., Sun W., Chen L., Tian S., Zhu L., Meng C., Zheng Z., Wang W.: What’s behind the mask: Understanding masked graph modeling for graph autoencoders. In: Proceedings of the 29th ACM SIGKDD Conference on Knowledge Discovery and Data Mining, pp. 1268–1279 (2023)

[14] Bender E.M., Gebru T., McMillan-Major A., Shmitchell S.: On the dangers of stochastic parrots: Can language models be too big? In: Proceedings of the 2021 ACM Conference on Fairness, Accountability, and Transparency, pp. 610–623 (2021)

[15] Dong Z., Zhang M., Payne P.R., Province M.A., Cruchaga C., Zhao T., Li F., Chen Y.: Rethinking the power of graph canonization in graph representation learning with stability. arXiv preprint arXiv:2309.00738 (2023)

[16] Abboud R., Ceylan I.I., Grohe M., Lukasiewicz T.: The surprising power of graph neural networks with random node initialization. arXiv preprint arXiv:2010.01179 (2020)

[17] Zhang H., Huang D., Chen Y., Li F.: Graphseqlm: A unified graph language framework for omic graph learning. arXiv preprint arXiv:2412.15790 (2024)

[18] Zhang H., Huang D., Li W., Province M., Chen Y., Payne P., Li F.: Galax: Graph-augmented language model for explainable reinforcement-guided subgraph reasoning in precision medicine. arXiv preprint arXiv:2509.20935 (2025)

[19] Persad S., Choo Z.-N., Dien C., Sohail N., Masilionis I., Chaligné R., Nawy T., Brown C.C., Sharma R., Pe’er I., : Seacells infers transcriptional and epigenomic cellular states from single-cell genomics data. Nature Biotechnology 41(12), 1746–1757 (2023) 10.1038/s41587-023-01716-9 PMC10713451 36973557

[20] Edgar R., Domrachev M., Lash A.E.: Gene expression omnibus: Ncbi gene expression and hybridization array data repository. Nucleic acids research 30(1), 207–210 (2002) 11752295 10.1093/nar/30.1.207 PMC99122

[21] Tarhan L., Bistline J., Chang J., Galloway B., Hanna E., Weitz E.: Single cell portal: an interactive home for single-cell genomics data. BioRxiv (2023)

[22] Regev A., Teichmann S.A., Lander E.S., Amit I., Benoist C., Birney E., Bodenmiller B., Campbell P., Carninci P., Clatworthy M., : The human cell atlas. elife 6, 27041 (2017) 10.7554/eLife.27041 PMC5762154 29206104

[23] Zhang H., Liang S., Xu T., Li W., Huang D., Dong Y., Li G., Miller J.P., Goedegebuure S.P., Sardiello M., : Biomedgraphica: An all-in-one platform for biomedical prior knowledge and omic signaling graph generation. bioRxiv, 2024–12 (2024) 10.1093/bioinformatics/btag355 PMC13281933 42246943

[24] Diehl A.D., Meehan T.F., Bradford Y.M., Brush M.H., Dahdul W.M., Dougall D.S., He Y., Osumi-Sutherland D., Ruttenberg A., Sarntivijai S., : The cell ontology 2016: enhanced content, modularization, and ontology interoperability. Journal of biomedical semantics 7(1), 44 (2016) 27377652 10.1186/s13326-016-0088-7 PMC4932724

[25] Zhang Y., Parmigiani G., Johnson W.E.: Combat-seq: batch effect adjustment for rna-seq count data. NAR genomics and bioinformatics 2(3), 078 (2020) 10.1093/nargab/lqaa078 PMC7518324 33015620

[26] Behdenna A., Colange M., Haziza J., Gema A., Appé G., Azencott C.-A., Nordor A.: pycombat, a python tool for batch effects correction in high-throughput molecular data using empirical bayes methods. BMC bioinformatics 24(1), 459 (2023) 38057718 10.1186/s12859-023-05578-5 PMC10701943

[27] Zhang D., Zhang W., He B., Zhang J., Qin C., Yao J.: Dnagpt: a generalized pretrained tool for multiple dna sequence analysis tasks. bioRxiv, 2023–07 (2023)

[28] Ferruz N., Schmidt S., Höcker B.: Protgpt2 is a deep unsupervised language model for protein design. Nature communications 13(1), 4348 (2022) 10.1038/s41467-022-32007-7 PMC9329459 35896542

[29] Rumelhart D.E., Hinton G.E., Williams R.J.: Learning representations by back-propagating errors. nature 323(6088), 533–536 (1986)

[30] Hornik K., Stinchcombe M., White H.: Multilayer feedforward networks are universal approximators. Neural networks 2(5), 359–366 (1989)

[31] Kipf T.N., Welling M.: Semi-supervised classification with graph convolutional networks. arXiv preprint arXiv:1609.02907 (2016)

[32] Veličković P., Cucurull G., Casanova A., Romero A., Lio P., Bengio Y.: Graph attention networks. arXiv preprint arXiv:1710.10903 (2017)

[33] Shi Y., Huang Z., Feng S., Zhong H., Wang W., Sun Y.: Masked label prediction: Unified message passing model for semi-supervised classification. arXiv preprint arXiv:2009.03509 (2020)

[34] Zhang H., Goedegebuure S.P., Ding L., DeNardo D., Fields R.C., Province M., Chen Y., Payne P., Li F.: M3netflow: A multi-scale multi-hop graph ai model for integrative multi-omic data analysis. iScience 28(3) (2025) 10.1016/j.isci.2025.111920 PMC11872513 40034855

[35] Zhang H., Cao D., Xu T., Chen E., Li G., Chen Y., Payne P., Province M., Li F.: Mosgraphflow: a novel integrative graph ai model mining signaling targets from multi-omic data. BMC Methods 2(1), 23 (2025) 41059350 10.1186/s44330-025-00041-8 PMC12497674

[36] Penzes P., VanLeeuwen J.-E.: Impaired regulation of synaptic actin cytoskeleton in alzheimer’s disease. Brain research reviews 67(1–2), 184–192 (2011) 21276817 10.1016/j.brainresrev.2011.01.003 PMC3109125

[37] Pelucchi S., Stringhi R., Marcello E.: Dendritic spines in alzheimer’s disease: how the actin cytoskeleton contributes to synaptic failure. International journal of molecular sciences 21(3), 908 (2020) 32019166 10.3390/ijms21030908 PMC7036943

[38] Jiang G., Xie G., Li X., Xiong J.: Cytoskeletal proteins and alzheimer’s disease pathogenesis: Focusing on the interplay with tau pathology. Biomolecules 15(6), 831 (2025) 40563471 10.3390/biom15060831 PMC12190275

[39] Stutzmann G.E.: Calcium dysregulation, ip3 signaling, and alzheimer’s disease. The Neuroscientist 11(2), 110–115 (2005) 15746379 10.1177/1073858404270899

[40] Ghosh A., Giese K.P.: Calcium/calmodulin-dependent kinase ii and alzheimer’s disease. Molecular brain 8(1), 78 (2015) 26603284 10.1186/s13041-015-0166-2 PMC4657223

[41] Crossley C.A., Rajani V., Yuan Q.: Modulation of l-type calcium channels in alzheimer’s disease: A potential therapeutic target. Computational and Structural Biotechnology Journal 21, 11–20 (2023) 36514335 10.1016/j.csbj.2022.11.049 PMC9719069

[42] Liu J., Chang L., Song Y., Li H., Wu Y.: The role of nmda receptors in alzheimer’s disease. Frontiers in neuroscience 13, 43 (2019) 30800052 10.3389/fnins.2019.00043 PMC6375899

[43] Lanskey J.H., Jafarian A., Hughes L.E., Karadag M., Kocagoncu E., Rouse M.A., Adams N.E., Naessens M., Raymont V., Woolrich M., : Alzheimer’s disease and memantine effects on nmda-receptor blockade: non-invasive in vivo insights from magnetoencephalography. Molecular Psychiatry, 1–10 (2025) 10.1038/s41380-025-03288-3 PMC12916295 41073581

[44] Kelly B.L., Ferreira A.: Beta-amyloid disrupted synaptic vesicle endocytosis in cultured hippocampal neurons. Neuroscience 147(1), 60–70 (2007) 17499934 10.1016/j.neuroscience.2007.03.047 PMC1993833

[45] Perdigão C., Barata M.A., Araújo M.N., Mirfakhar F.S., Castanheira J., Guimas Almeida C.: Intracellular trafficking mechanisms of synaptic dysfunction in alzheimer’s disease. Frontiers in cellular neuroscience 14, 72 (2020) 32362813 10.3389/fncel.2020.00072 PMC7180223

[46] Zhang W., Xu C., Sun J., Shen H.-M., Wang J., Yang C.: Impairment of the autophagy–lysosomal pathway in alzheimer’s diseases: pathogenic mechanisms and therapeutic potential. Acta Pharmaceutica Sinica B 12(3), 1019–1040 (2022) 35530153 10.1016/j.apsb.2022.01.008 PMC9069408

[47] Nixon R.A., Rubinsztein D.C.: Mechanisms of autophagy–lysosome dysfunction in neurodegenerative diseases. Nature Reviews Molecular Cell Biology 25(11), 926–946 (2024) 39107446 10.1038/s41580-024-00757-5 PMC12239022

[48] Li Z., Yin B., Zhang S., Lan Z., Zhang L.: Targeting protein kinases for the treatment of alzheimer’s disease: recent progress and future perspectives. European Journal of Medicinal Chemistry 261, 115817 (2023) 37722288 10.1016/j.ejmech.2023.115817

[49] Wu X., Yang Z., Zou J., Gao H., Shao Z., Li C., Lei P.: Protein kinases in neurodegenerative diseases: current understandings and implications for drug discovery. Signal Transduction and Targeted Therapy 10(1), 146 (2025) 40328798 10.1038/s41392-025-02179-x PMC12056177

[50] Thompson J.C., Davis C., Deshpande C., Hwang W.-T., Jeffries S., Huang A., Mitchell T.C., Langer C.J., Albelda S.M.: Gene signature of antigen processing and presentation machinery predicts response to checkpoint blockade in non-small cell lung cancer (nsclc) and melanoma. Journal for immunotherapy of cancer 8(2), 000974 (2020) 10.1136/jitc-2020-000974 PMC7542663 33028693

[51] Kubo T., Asano S., Sasaki K., Murata K., Kanaseki T., Tsukahara T., Hirohashi Y., Torigoe T.: Assessment of cancer cell-expressed hla class i molecules and their immunopathological implications. HLA 103(5), 15472 (2024) 10.1111/tan.15472 38699870

[52] Zeng Y., Lv X., Du J.: Natural killer cell-based immunotherapy for lung cancer: Challenges and perspectives. Oncology Reports 46(5), 1–14 (2021) 10.3892/or.2021.8183 PMC8444189 34498710

[53] Mandai M., Hamanishi J., Abiko K., Matsumura N., Baba T., Konishi I.: Dual faces of ifn*γ* in cancer progression: a role of pd-l1 induction in the determination of pro-and antitumor immunity. Clinical cancer research 22(10), 2329–2334 (2016) 27016309 10.1158/1078-0432.CCR-16-0224

[54] Lingling Z., Jiewei L., Li W., Danli Y., Jie Z., Wen L., Dan P., Lei P., Qinghua Z.: Molecular regulatory network of pd-1/pd-l1 in non-small cell lung cancer. Pathology-Research and Practice 216(4), 152852 (2020) 32057515 10.1016/j.prp.2020.152852

[55] Hirayama A., Tanaka K., Tsutsumi H., Nakanishi T., Yamashita S., Mizusaki S., Ishii Y., Ota K., Yoneshima Y., Iwama E., : Regulation of pd-l1 expression in non–small cell lung cancer by interleukin-1β. Frontiers in immunology 14, 1192861 (2023) 37441079 10.3389/fimmu.2023.1192861 PMC10333574

[56] Ntellas P., Mavroeidis L., Gkoura S., Gazouli I., Amylidi A.-L., Papadaki A., Zarkavelis G., Mauri D., Karpathiou G., Kolettas E., : Old player-new tricks: non angiogenic effects of the vegf/vegfr pathway in cancer. Cancers 12(11), 3145 (2020) 33121034 10.3390/cancers12113145 PMC7692709

[57] Ghalehbandi S., Yuzugulen J., Pranjol M.Z.I., Pourgholami M.H.: The role of vegf in cancer-induced angiogenesis and research progress of drugs targeting vegf. European Journal of Pharmacology 949, 175586 (2023) 36906141 10.1016/j.ejphar.2023.175586

[58] Lee C., Kim M.-J., Kumar A., Lee H.-W., Yang Y., Kim Y.: Vascular endothelial growth factor signaling in health and disease: from molecular mechanisms to therapeutic perspectives. Signal Transduction and Targeted Therapy 10(1), 170 (2025) 40383803 10.1038/s41392-025-02249-0 PMC12086256

[59] Wang T., Shao W., Huang Z., Tang H., Zhang J., Ding Z., Huang K.: Mogonet integrates multi-omics data using graph convolutional networks allowing patient classification and biomarker identification. Nature communications 12(1), 3445 (2021) 10.1038/s41467-021-23774-w PMC8187432 34103512

[60] Dong Z., Zhao Q., Payne P.R., Province M.A., Cruchaga C., Zhang M., Zhao T., Chen Y., Li F.: Highly accurate disease diagnosis and highly reproducible biomarker identification with pathformer. Research Square, 3 (2023)

[61] Zhang H., Chen Y., Payne P., Li F.: Using deepsignalingflow to mine signaling flows interpreting mechanism of synergy of cocktails. npj Systems Biology and Applications 10(1), 92 (2024) 39169016 10.1038/s41540-024-00421-w PMC11339460

[62] Yao Z., van Velthoven C.T., Kunst M., Zhang M., McMillen D., Lee C., Jung W., Goldy J., Abdelhak A., Aitken M., : A high-resolution transcriptomic and spatial atlas of cell types in the whole mouse brain. Nature 624(7991), 317–332 (2023) 38092916 10.1038/s41586-023-06812-z PMC10719114

[63] Abdi H., Williams L.J.: Principal component analysis. Wiley interdisciplinary reviews: computational statistics 2(4), 433–459 (2010)

[64] Peterson L.E.: K-nearest neighbor. Scholarpedia 4(2), 1883 (2009)

[65] U.S. National Library of Medicine: Age Groups - Medical Subject Headings (MeSH). https://www.ncbi.nlm.nih.gov/mesh/68009273. Accessed: 2025–10-09 (1999)

