# Supplementary material for: OmniCellTOSG: The First Cell Text-Omic Signaling Graphs Dataset for Graph Language Foundation Modeling
Source: Res Sq. 2026 Feb 17:rs.3.rs-8774770. Preprint. [Version 1] doi: 10.21203/rs.3.rs-8774770/v1 (PMC12934919; doi:10.21203/rs.3.rs-8774770/v1)
Supplement: 1 [file NIHPPRS8774770V1-supplement-1.pdf]

5 Supplementary Notes

5.1 Data Collection

CZ CellxGene Database

Data from the CELLxGENE database were obtained using the CZI Science CELLxGENE Census Python API, with the census version fixed to `2025-01-30` and the organism set to `Homo sapiens`. We retrieved matrices directly in `.h5ad` (AnnData) format, together with standardized per-cell metadata fields, to facilitate downstream harmonization and provenance tracking.

Brain Cell Atlas

Human datasets were manually downloaded from the Brain Cell Atlas portal after applying the species filter `Human`. Because some processed matrices lack unique per-sample identifiers, we preserved the original project identifiers from the source datasets in the resulting `.h5ad` files. This ensures traceability across preprocessing steps and alignment with external references.

GEO Database and Others

We manually retrieved datasets hosted on NCBI GEO to complement CELLxGENE and Brain Cell Atlas with broader organ and disease coverage, larger cohort sizes for our analyses. Selection prioritized (i) availability of key annotations used for harmonization and balancing (e.g., `cell_type`, `disease`, `tissue/tissue_general`, `sex`, and `suspension_type`; `development_stage` when available), (ii) sufficient cell counts to support robust distribution matching and downstream modeling, and (iii) feasibility of standard quality-control procedures (empty-droplet removal, doublet detection, ambient RNA mitigation) from the provided raw or study-curated matrices. We additionally incorporated two curated non-GEO resources where they provide high-quality matrices that fill specific anatomical or disease gaps in our corpus: (i) Human Cell Atlas project matrices for pancreatic tissue and retained with their original project identifiers; and (ii) the hepatitisCatlas dataset from the Broad Single Cell Portal, which supplies a curated hepatitis C cohort with consistent annotation fields. These additions are limited and purpose-driven—used only to complete organ/disease coverage and to maintain uniform preprocessing and metadata standards across the integrated collection. Accession lists and download links are provided in Table S1.

5.2 Data Preprocessing

To support uniform downstream analysis across heterogeneous repositories, we standardized all sources to an AnnData saved as `.h5ad`. Datasets from GEO, the Human Cell Atlas, and a hepatitis atlas arrived in diverse encodings—including Matrix Market triplets (`barcodes.tsv.gz`, `features.tsv.gz`, `matrix.mtx.gz`), compressed CSV/TXT (`.csv.gz`/`.txt.gz`), and HDF5 (`.h5`)—whereas CellxGene and Brain Cell Atlas releases were largely pre-packaged as `.h5ad`. For non-`.h5ad` inputs, we reconciled barcodes, gene feature tables, and count matrices, then ingested the data with Scanpy to construct AnnData objects that preserve gene–cell mappings and available per-cell/per-gene annotations. We subsequently harmonized `obs` (cells) and `var` (genes) to a consistent schema aligned with CellxGene/Brain Cell Atlas conventions (standardized identifiers and controlled-vocabulary attributes for tissue,

disease, donor, sex, age; plus gene symbols/IDs and feature types), and exported the harmonized objects to `.h5ad` for consistent I/O.

Quality checks verified internal consistency between matrix dimensions and annotation tables, the presence and datatypes of required metadata fields, and read/write integrity of the final files. The resulting curated, schema-aligned `.h5ad` datasets serve as standardized inputs for meta-cell construction and subsequent transformation into OmniCellTOSG, enabling reproducible multi-study analyses under a unified data model.

5.3 `CellTOSG_Loader` Package

5.3.1 Package Usage

For instance, row-level metadata filters (`conditions`; required) defined the cohort task retrieval scope, such as tissue- and disease-level criteria (e.g., {`tissue_general`: brain, `disease_name`: Alzheimer's Disease}). The `task` and `label_column` fully specifies the cohort and label semantics for downstream modeling. As to the `sample_ratio`, this parameters will extracted this ratio of samples from whole candidate cells, since some files are pretty large and it will burst the memory storage. By using this ratio, we will sampling this ratio of cells from whole candidate cells. Following are the example code for using the package

Listing 1: Loading the CellTOSG dataset and extracting graph-based features

```
from CellTOSG_Loader import CellTOSGDataLoader

# --- Build loader (uses your argparse ‘args‘) ---
conditions = {
    "tissue_general": args.tissue_general,
    "disease_name": args.disease_name,   # or: "disease": args.disease_name
    # "suspension_type": args.suspension_type,
    # "cell_type": args.cell_type,
    # "sex": args.sex,
}

dataset = CellTOSGDataLoader(
    root=args.dataset_root,
    conditions=conditions,
    task=args.task,                          # "disease" / "sex" / "cell_type"
    label_column=args.label_column,          # "disease" / "sex" / "cell_type"
    sample_ratio=args.sample_ratio,          # mutually exclusive with sample_size
    sample_size=args.sample_size,
    shuffle=args.shuffle,
    stratified_balancing=args.stratified_balancing,
    extract_mode=args.extract_mode,          # "inference" / "train"
    train_text=args.train_text, # False -> return precomputed name/desc embeddings
    train_bio=args.train_bio,   # False -> return precomputed sequence embeddings
    correction_method=args.correction_method, # None / "combat_seq"
    output_dir=args.output_dir,
)

# --- Access tensors/arrays ---
if args.extract_mode == "inference":
    X = dataset.data                         # pandas.DataFrame (expression/features)
    y = dataset.labels                       # pandas.DataFrame
    metadata = dataset.metadata              # pandas.DataFrame (row-aligned metadata)
else:
    X = dataset.data                         # dict: {"train": X_train, "test": X_test}
    y = dataset.labels                       # dict: {"train": y_train, "test": y_test}
    metadata = dataset.metadata              # dict: {"train": meta_train, "test":
      meta_test}
```

```
all_edge_index = dataset.edge_index                    # full graph (COO [2, E])
internal_edge_index = dataset.internal_edge_index      # optional transcript-protein edges
ppi_edge_index = dataset.ppi_edge_index                # optional PPI edges
x_name_emb, x_desc_emb, x_bio_emb = pre_embed_text(args, dataset, pretrain_model, device) #
      Prepare text and seq embeddings
```

5.3.2 Stratified Retrieval Algorithm

Algorithm 1 Stratified Retrieval Algorithm

Input: Data $\mathcal{X}$; query q; config $\Lambda(\lambda) = (b, b_0, K, j^\star)$; tolerance δ; seed s; upsample flag P
Output: Balanced, stratified retrieval $\mathcal{X}_R$

Phase I: Query-Constrained Extraction
$R(q) \leftarrow \{\, x \in \mathcal{X} : \bigwedge_{(a,V_a)\in q} \mathcal{I}[u_a(x) \in V_a]\}$
Phase II: Config-Driven Balancing
CA $\leftarrow \{\, x \in R(q) : \; u_b(x) \neq b_0 \,\}$
$q_{-b} \leftarrow \{(a, V_a) \in q : \; a \neq b\}$
NM $\leftarrow \{\, x \in \mathcal{X} : \; u_b(x) = b_0 \; \wedge \; \bigwedge_{(a,V_a)\in q_{-b}} \mathcal{I}[u_a(x) \in V_a]\}$
drop rows in CA, NM with missing u_{k_i} for any $k_i \in K$
if $|\mathrm{CA}| \leq |\mathrm{NM}|$ **then**
  ref $\leftarrow$ CA; **tgt** $\leftarrow$ NM
else
  ref $\leftarrow$ NM; **tgt** $\leftarrow$ CA
end if
$\mathcal{X}_R \leftarrow \varnothing$
for all $\kappa \in \{\kappa_K(x) : \; x \in$ **ref**$\}$ **do**
  $\xi \leftarrow |\{\, x \in \textbf{ref} : \; \kappa_K(x) = \kappa \,\}|$
  Match $\leftarrow \varnothing$
  Used $\leftarrow \varnothing$
  for $t = 0$ **to** δ **do**
    $\mathrm{Cand}_t \leftarrow \{\, x \in \textbf{tgt} : \; u_{k_i}(x) = \kappa_i \; \forall i \neq j^\star, \; d_{k^\star}(u_{k^\star}(x), \kappa_{j^\star}) \leq t \,\} \setminus \mathrm{Used}$
    $\eta \leftarrow \min\{\, \xi - |\mathrm{Match}|, \; |\mathrm{Cand}_t| \,\}$
    sample η **w/o replacement** from Cand_t into Take
    Match $\leftarrow$ Match $\cup$ Take
    Used $\leftarrow$ Used $\cup$ idx(Take)
    if $|\mathrm{Match}| = n_\kappa$ **then**
      break
    end if
  end for
  if $0 < |\mathrm{Match}| < n_\kappa$ **and** $P = \mathrm{TRUE}$ **then**
    Match $\leftarrow (n_\kappa - |\mathrm{Match}|)$ draws of samples (with replacement) from Match
  end if
  if $|\mathrm{Match}| > 0$ **then**
    $\mathcal{X}_R \leftarrow \{x \in \textbf{ref} : \; \kappa_K(x) = \kappa\} \cup \mathrm{Match}$
  end if
end for
$Y_R \leftarrow \{\; \ell_b(x) \; : \; x \in \mathcal{X}_R \;\}$
return $\mathcal{X}_R$, Y_R

5.3.3 Train-test Datasets Split

In order to avoid subject-level data leakage, we performed a donor-level split in which all samples from the same donor were assigned exclusively to either the training set or the test set. A unique donor identifier was constructed by combining the study identifier with the donor identifier, ensuring that donors remain distinct even when the same donor label may appear in different studies. For each cohort, we selected a set of test donors to achieve an approximately fixed test set size at the sample level, while imposing an explicit upper bound to prevent the test set from being dominated by donors contributing unusually large numbers of samples. Donors were

processed in a randomized order with a preference for smaller donors to better match the intended test proportion.

5.4 External Dataset

We used a Brain Cell Atlas preprocessed GSE129308 AnnData (H5AD) file as an external Alzheimer's disease (AD) cohort and applied a unified preprocessing pipeline across disease, sex, and cell type prediction tasks. Cells lacking donor identifiers or cell type annotations were excluded, and disease and sex labels were curated by removing unknown or unannotated entries and normalizing them into binary targets (control versus AD for disease, female versus male for sex). Train and test sets were defined at the donor level to eliminate data leakage, ensuring that all cells from a given donor were assigned exclusively to a single split. For binary tasks (disease and sex), donors were selected based on cell counts to match the desired test fraction while approximately preserving label prevalence under a strict cap on test set size, whereas for cell type prediction donors were partitioned solely according to cell counts. Consistent label coverage across splits was maintained by retaining only the intersection of cell types shared between training and test sets. Class imbalance was addressed through controlled downsampling to obtain approximately balanced class distributions in both partitions. The processed H5AD files were subsequently converted into fixed width NumPy expression matrices by mapping gene identifiers (gene symbols or Ensembl IDs) to a predefined BioMedGraphica feature index space, with unmapped features zero padded. Expression values were library size normalized to a fixed target sum and log1p transformed. Labels were exported as encoded NumPy arrays together with mapping tables to ensure reproducibility. The resulting feature matrices and label vectors are directly compatible with the input requirements of CellTOSG-FM for downstream modeling.

6 Supplementary Tables

Table S1: Detailed information on datasets collected from GEO and other sources

Platform	Dataset ID	Disease(s)	Tissue General	Cell Count	Links
GEO	GSE183852	Dilated cardiomyopathy	Heart	49 723	https://www.ncbi.nlm.nih.gov/geo/query/acc.cgi?acc=GSE183852
GEO	GSE125449	Hepatocellular carcinoma; Intrahepatic Cholangiocarcinoma	Liver	9946	https://www.ncbi.nlm.nih.gov/geo/query/acc.cgi?acc=GSE125449
Single Cell Portal	hepatitisCatlas	Hepatitis C	Liver, Blood	8350	https://singlecell.broadinstitute.org/single_cell/study/SCP2407/single-cell-atlas-of-the-liver-myeloid-compartment-before-and-after-cure-of-chronic-viral-hepatitis#study-download
Human Cell Atlas	PancreasTopographiesTosti10x	Carcinosarcoma; Chronic Pancreatitis; Pancreatic Ductal Adenocarcinoma; Pancreatic Neuroendocrine Neoplasm	Pancreas	125 757	https://explore.data.humancellatlas.org/projects/b3938158-4e8d-4fdb-9e13-9e94270dde16/project-matrices

Table S2: Description of the final attribute set $\mathcal{A}$.

Attribute	Meaning	Type	Req.	Example
source	Origin of the record or data repository/provider.	string	No	CellxGene, BrainCellAtlas
dataset_id	Unique dataset identifier within/among sources.	string	Yes	GSE144744, EGAS00001004107
suspension_type	sc/snRNA-seq.	string	Yes	cell, nucleus
tissue_general (coarse-grained)	Tissue/organ category.	string	Yes*	brain, liver
tissue (fine-grained)	Specific tissue/region.	string	No	prefrontal cortex, heart right ventricle
matrix_file_path	File relative path to the expression matrix file.	string	Yes	/expression_matrix/braincellatlas/brain_part_0.npy
matrix_row_idx	Row index in the matrix for this entity/sample.	integer	Yes	2025
donor_id	Donor ID.	string	Yes	Donor26
CMT_id	Cell type ID mapped to Cell Ontology.	string	No	CMT0151
CMT_name	Cell type name mapped to Cell Ontology.	string	No	microglial cell
disease_BMG_name	Standardized disease label mapped to BioMedGraphica terminology.	string	Yes*	Alzheimer's disease
disease_BMG_id	Standardized disease ID mapped to BioMedGraphica ID.	string	Yes*	BMGC_DS00092
development_stage_category	Broad development stage category.	enum	No	embryo, fetal, adult
sex_normalized	Normalized sex label after harmonization.	enum	Yes	male, female, unknown

Notes: "Req." = required field. Yes = mandatory; No = optional; Yes* = required in most cases (e.g., for healthy/control, age & sex cohorts).

Table S3: OmniCellTOSG Dataset Overview and Detailed Statistics

Diseases	Organ/Tissue Types	# of Original Cells	# of Meta Cells
Normal	Multiple Tissue*	56,283,477	280,978
Covid-19	Blood, Brain, Digestive System, Lung, Nose, Respiratory System, Saliva	5,130,024	25,621
Parkinson Disease	Brain	1,736,202	8,675
Alzheimer's Disease	Brain	1,665,182	8,316
Glioblastoma	Brain	1,236,376	6,171
Dementia	Brain	1,052,021	5,259
Malignant Ovarian Serous Tumor	Abdomen, Colon, Fallopian Tube, Intestine, Large Intestine, Liver, Lymph Node, Musculature, Omentum, Ovary, Paracolic Gutter, Urinary Bladder, Uterus	927,205	4,629
Lung Adenocarcinoma	Adrenal Gland, Brain, Liver, Lung, Lymph Node, Pleural Fluid	862,494	4,304
Systemic Lupus Erythematosus	Blood	777,258	3,886
Crohn Disease	Colon, Small Intestine	572,174	2,844
Breast Cancer	Axilla, Brain, Breast, Chest Wall, Liver, Lung, Neck, Skeletal System, Skin Of Body	553,514	2,760
Dilated Cardiomyopathy	Heart	519,709	2,596
Multiple Sclerosis	Blood, Brain	442,149	2,210
Chronic Kidney Disease	Kidney	370,831	1,851
Amyotrophic Lateral Sclerosis	Brain	321,851	1,608
Atrial Fibrillation	Heart	273,963	1,369
Atherosclerosis	Heart, Vasculature	259,721	1,297
Frontotemporal Dementia	Brain	252,352	1,260
Temporal Lobe Epilepsy	Brain	246,167	1,230
Squamous Cell Lung Carcinoma	Lung	243,071	1,214
Pulmonary Fibrosis	Lung	218,932	1,094
Nonpapillary Renal Cell Carcinoma	Adrenal Gland, Kidney, Vasculature	207,496	1,035
Acute Kidney Failure	Kidney	189,295	944
Clear Cell Renal Carcinoma	Blood, Kidney, Lymph Node	187,792	938
B-cell Acute Lymphoblastic Leukemia	Blood	183,023	913
Triple-negative Breast Carcinoma	Breast, Exocrine Gland, Pleural Fluid	179,724	897
Gliomas	Brain	169,399	845
Chronic Obstructive Pulmonary Disease	Lung, Respiratory System	164,361	820
Myocardial Infarction	Brain, Heart	152,055	760
Epilepsy	Brain	134,460	671
Follicular Lymphoma	Lymph Node	122,702	613
Non-small Cell Lung Carcinoma	Lung	120,796	603
Pancreatic Ductal Adenocarcinoma	Pancreas	118,676	592
Progressive Supranuclear Palsy	Brain	117,482	585
Basal Cell Carcinoma	Skin Of Body	115,456	573
Unknown	Blood	109,488	547
Primary Sclerosing Cholangitis	Liver	104,667	522
Arrhythmogenic Right Ventricular Cardiomyopathy	Heart	104,496	522
Unclassified	Brain	99,009	494
Pick Disease	Brain	98,043	489
Post-covid-19 Disorder	Blood	97,224	486
Respiratory System Disorder	Blood	94,987	474
Digestive System Disorder	Small Intestine	89,849	449
Estrogen-receptor Positive Breast Cancer	Breast	87,648	438
Invasive Ductal Breast Carcinoma	Breast, Exocrine Gland	86,873	433
Juvenile Dermatomyositis	Blood	82,666	413
Small Cell Lung Carcinoma	Adrenal Gland, Axilla, Brain, Liver, Lung, Lymph Node, Pleural Fluid	79,040	390
Amyotrophic Lateral Sclerosis 26 With Or Without Frontotemporal Dementia	Brain	73,797	368
Interstitial Lung Disease	Lung	68,456	342
Benign Prostatic Hyperplasia	Prostate Gland	66,181	330
Lewy Body Dementia	Brain	65,789	327
Common Variable Immunodeficiency	Blood	64,081	319
B-cell Non-hodgkin Lymphoma	Bone Marrow	59,746	298
Blastoma	Liver	57,445	287
Opiate Dependence	Brain	54,399	271
Autism Spectrum Disorder	Brain	52,003	260

Diseases	Organ/Tissue Types	# of Original Cells	# of Meta Cells
Oropharynx Squamous Cell Carcinoma	Digestive System	50,000	250
Rheumatoid Arthritis	Blood	48,637	243
Clonal Hematopoiesis	Blood	47,354	236
Luminal B Breast Carcinoma	Pleural Fluid	46,128	229
Sjogren Syndrome	Exocrine Gland	45,231	226
Major Depressive Disorder	Brain	41,944	209
Primary Biliary Cholangitis	Liver	39,994	199
Periodontitis	Mucosa	38,520	192
Influenza	Blood, Brain	34,549	171
Pilocytic Astrocytoma	Brain	34,291	171
Crohn Ileitis	Small Intestine	32,458	162
Pneumonia	Lung	31,923	159
Pulmonary Emphysema	Lung	31,792	158
Diffuse Large B-cell Lymphoma	Lymph Node, Respiratory System, Small Intestine	31,131	153
Chronic Rhinitis	Nose	29,137	145
Listeriosis	Placenta	28,237	141
Oral Cavity Squamous Cell Carcinoma	Digestive System	28,186	140
Toxoplasmosis	Placenta	28,098	140
Plasmodium Malariae Malaria	Placenta	27,958	139
Acute Myeloid Leukemia	Bone Marrow	27,852	139
Gastric Intestinal Metaplasia	Stomach	27,462	136
Gastritis	Stomach	26,639	133
Hiv Infectious Disease	Blood	24,548	122
Type 1 Diabetes Mellitus	Pancreas	22,400	112
Lung Large Cell Carcinoma	Lung	21,167	105
Tubular Adenoma	Colon	20,442	99
Luminal A Breast Carcinoma	Pleural Fluid	20,403	101
Type 2 Diabetes Mellitus	Kidney, Vasculature	19,262	95
Leukoencephalopathy, Diffuse Hereditary, With Spheroids 1	Brain	19,164	93
Idiopathic Parkinson's Disease	Brain	19,002	95
Hydrosalpinx	Fallopian Tube	17,798	88
Cystic Fibrosis	Lung	17,590	87
Trisomy 18	Brain	16,900	84
Down Syndrome	Bone Marrow	16,743	83
Invasive Lobular Breast Carcinoma	Breast, Exocrine Gland	16,507	81
Her2 Positive Breast Carcinoma	Breast	16,017	80
Squamous Cell Carcinoma	Skin Of Body	15,328	75
Secondary Progressive Multiple Sclerosis	Brain	14,469	72
Cell Stress	Brain	13,165	65
Melanoma	Skin Of Body	13,141	65
Kidney Oncocytoma	Kidney	12,610	63
Lymphangioleiomyomatosis	Lung	12,374	61
Relapsing-remitting Multiple Sclerosis	Brain	12,201	61
Non-compaction Cardiomyopathy	Heart	11,632	57
Adenocarcinoma	Colon, Large Intestine, Small Intestine	11,483	55
Intrahepatic Cholangiocarcinoma	Liver	11,466	57
Barrett Esophagus	Esophagus	10,952	54
Metastatic Melanoma	Brain	10,895	54
Colon Sessile Serrated Adenoma/polyp	Colon, Intestine	10,893	53
Pleomorphic Carcinoma	Lung	10,765	53
Hypersensitivity Pneumonitis	Lung	10,379	51
Aspiration Pneumonia	Brain	10,204	51
Non-specific Interstitial Pneumonia	Lung	8,597	42
Acute Myocardial Infarction	Brain	8,033	40
Carcinosarcoma	Pancreas	7,967	39
Hepatitis C Virus Infection	Liver	7,607	38
Breast Carcinoma	Breast	7,373	36
Hepatocellular Carcinoma	Liver	7,165	35
Primary Cutaneous Diffuse Large B-cell Lymphoma, Leg Type	Skin Of Body	7,097	35
Tubulovillous Adenoma	Colon, Intestine	6,793	32
Gingivitis	Mucosa	6,587	32
Injury	Skin Of Body	5,983	29
Mixed Gliomas	Brain	5,979	29
Anencephaly	Lung	5,499	27
Pancreatic Neuroendocrine Neoplasm	Pancreas	5,233	26

**Diseases**	**Organ/Tissue Types**	**# of Original Cells**	**# of Meta Cells**
Congenital Heart Disease	Brain	5,046	25
Pulmonary Sarcoidosis	Lung	4,886	24
Wilms Tumor	Kidney	4,636	23
Heart Failure	Brain	4,594	22
Acute Promyelocytic Leukemia	Bone Marrow	3,734	18
Pulpitis	Skeletal System	3,655	18
Respiratory Failure	Digestive System	3,335	16
Anaplastic Astrocytoma	Brain	3,097	15
Macular Degeneration	Eye	3,011	15
Tongue Cancer	Brain	2,992	14
Mild Cognitive Impairment	Brain	2,851	14
Chronic Pancreatitis	Pancreas	2,666	13
Neuroendocrine Carcinoma	Small Intestine	2,623	13
Hyperplastic Polyp	Colon	2,616	13
Chromophobe Renal Cell Carcinoma	Kidney	2,576	12
Long Covid-19	Digestive System	2,306	11
Colorectal Cancer	Colon	2,199	10
Malignant Pancreatic Neoplasm	Brain	2,148	10
Enamel Caries	Skeletal System	2,015	10
Heart Disorder	Brain	1,957	9
Cataract	Eye	1,810	9
**Total**		79,195,364	395,317

* *Multiple Tissue (Normal):* Adipose Tissue, Adrenal Gland, Bladder Organ, Blood, Bone Marrow, Brain, Breast, Central Nervous System, Colon, Cortex, Digestive System, Embryo, Endocrine Gland, Esophagogastric Junction, Esophagus, Exocrine Gland, Eye, Fallopian Tube, Forelimb, Gallbladder, Head, Heart, Hindlimb, Immune System, Intestine, Kidney, Lamina Propria, Large Intestine, Liver, Lung, Lymph Node, Milk, Mucosa, Musculature, Nose, Omentum, Ovary, Pancreas, Placenta, Pleura, Prostate Gland, Respiratory System, Scalp, Sensory System, Skeletal System, Skin Of Body, Small Intestine, Spinal Cord, Spleen, Stomach, Tendon Of Semitendinosus, Testis, Tongue, Ureter, Urinary Bladder, Uterus, Vasculature, Yolk Sac

Table S4: Model performance across diseases on the cell-type annotation task

Disease	Metric	GCN	GAT	UniMP	DNN	scCELLO	scFoundation	scGPT	CellTOSG-FM
**AD**	Accuracy	0.2353±0.1544	0.0537±0.0107	0.4404±0.0279	0.7145±0.0334	0.7788±0.0164	0.7719±0.0264	0.7944±0.0108	**0.8792±0.0164**
	F1	0.0394±0.0236	0.0113±0.0021	0.0747±0.0089	0.3108±0.0286	0.5669±0.0258	0.4486±0.0631	0.4234±0.0260	**0.6997±0.0838**
**LUAD**	Accuracy	0.0733±0.0085	0.0583±0.0047	0.0700±0.0187	0.4333±0.0306	0.2067±0.0368	0.5133±0.0266	0.2467±0.0978	**0.5767±0.0628**
	F1	0.0175±0.0020	0.0155±0.0027	0.0112±0.0051	0.3615±0.0366	0.1649±0.0355	0.4489±0.0402	0.2057±0.1082	**0.5247±0.0672**
**AF**	Accuracy	0.1810±0.0488	0.1732±0.0203	0.2500±0.0000	0.8242±0.0096	0.8737±0.0103	0.8802±0.0488	0.8112±0.0112	**0.9792±0.0018**
	F1	0.0405±0.0075	0.0368±0.0037	0.0500±0.0000	0.6079±0.0367	0.7431±0.0173	0.8391±0.0942	0.7078±0.0220	**0.9744±0.0063**
**SLE**	Accuracy	0.1383±0.0230	0.2482±0.0591	0.2801±0.0779	0.7305±0.0887	0.5674±0.0509	0.8475±0.0558	0.8227±0.1248	**0.8972±0.0929**
	F1	0.0342±0.0042	0.0905±0.0278	0.0978±0.0435	0.6678±0.1201	0.3990±0.0629	0.8449±0.0615	0.8111±0.1415	**0.8617±0.1463**

Table S5: Performance of models on cell conditions (disease vs. normal) across diseases

Disease	Metric	GCN	GAT	UniMP	DNN	CellTOSG-FM
**AD**	Accuracy	0.5045±0.0064	0.4864±0.0099	0.5407±0.0289	0.6957±0.0330	**0.7913±0.0147**
	F1	0.3928±0.0841	0.3821±0.0635	0.4697±0.0968	0.6909±0.0366	**0.7894±0.0151**
**LUAD**	Accuracy	0.5774±0.0539	0.6730±0.0346	0.7111±0.0299	0.7972±0.0132	**0.8400±0.0152**
	F1	0.4952±0.1167	0.6472±0.0442	0.7019±0.0374	0.7902±0.0134	**0.8381±0.0150**
**AF**	Accuracy	0.5231±0.0327	0.5046±0.0065	0.4884±0.0657	0.6944±0.0354	**0.9653±0.0204**
	F1	0.4116±0.1107	0.3470±0.0193	0.3931±0.0595	0.6826±0.0366	**0.9653±0.0204**
**SLE**	Accuracy	0.5556±0.0109	0.5983±0.1013	0.5940±0.0757	0.8397±0.1180	**0.9979±0.0030**
	F1	0.4199±0.0877	0.5250±0.1531	0.5498±0.0712	0.8265±0.1339	**0.9978±0.0031**

Table S6: Performance of models on cell sex classifications

Disease	Metric	GCN	GAT	UniMP	DNN	CellTOSG-FM
**AD**	Accuracy	0.5098±0.0100	0.5020±0.0028	0.5059±0.0144	0.6588±0.0546	**0.7882±0.0144**
	F1	0.3730±0.0411	0.3409±0.0107	0.4359±0.0459	0.6456±0.0624	**0.7864±0.0147**
**AF**	Accuracy	0.5580±0.0256	0.5072±0.0489	0.4529±0.0505	0.4964±0.0051	**0.9022±0.0307**
	F1	0.4795±0.0704	0.4401±0.0632	0.3998±0.0540	0.3317±0.0023	**0.9019±0.0307**

Table S7: Model performances on cell sex classfication across age groups in AF

Age Group	Metric	GCN	GAT	UniMP	DNN	CellTOSG-FM
**Aged (≥ 65yrs)**	Accuracy	0.5897±0.0363	0.5385±0.1088	0.4359±0.0725	0.5385±0.1088	**0.6410±0.0725**
	F1	0.6025±0.1143	0.4996±0.3534	0.3762±0.2882	0.5079±0.3592	**0.7593±0.0429**
**80 and over**	Accuracy	0.5527±0.0332	0.5021±0.0418	0.4557±0.0474	0.4895±0.0215	**0.9451±0.0239**
	F1	0.4533±0.1468	0.4603±0.2409	0.3668±0.2227	0.4292±0.3035	**0.9436±0.0261**

Table S8: Model performances on sex classfication across cell types in AF

Cell Type	Metric	GCN	GAT	UniMP	DNN	CellTOSG-FM
Cardiac muscle cell	Accuracy	0.6333±0.0943	0.4333±0.0624	0.3833±0.0624	0.5000±0.0000	**0.9000±0.0707**
	F1	0.6210±0.0390	0.3028±0.2421	0.2451±0.2525	0.4444±0.3143	**0.9076±0.0633**
Cardiac blood vessel endothelial cell	Accuracy	0.5000±0.0000	0.4444±0.0786	0.4630±0.0262	0.5000±0.0000	**1.0000±0.0000**
	F1	0.2222±0.3143	0.4921±0.2469	0.4274±0.3029	0.4444±0.3143	**1.0000±0.0000**
Fibroblast	Accuracy	0.4815±0.0262	0.5185±0.0944	0.4259±0.0944	0.5000±0.0000	**0.8519±0.0262**
	F1	0.2222±0.3143	0.5221±0.2606	0.3932±0.2239	0.4444±0.3143	**0.8677±0.0150**
Adipocyte	Accuracy	0.6333±0.0943	0.6000±0.0816	0.5000±0.0000	0.5000±0.0000	**0.9000±0.0000**
	F1	0.7350±0.0483	0.4091±0.3038	0.1481±0.2095	0.4444±0.3143	**0.9091±0.0000**
Mesothelial cell	Accuracy	0.7083±0.1559	0.6667±0.2357	0.6250±0.1021	0.5000±0.0000	**1.0000±0.0000**
	F1	0.7302±0.0898	0.5556±0.4157	0.3556±0.2740	0.4444±0.3143	**1.0000±0.0000**
Others*	Accuracy	0.5000±0.0000	0.5185±0.0262	0.4444±0.0454	0.4815±0.0262	**0.8148±0.1048**
	F1	0.2222±0.3143	0.5136±0.2348	0.3961±0.2863	0.4274±0.3029	**0.8185±0.1075**

Others*: Endocardial cell; Lymphocyte; Macrophage; Pericyte; Schwann cell

7 Supplementary Figures

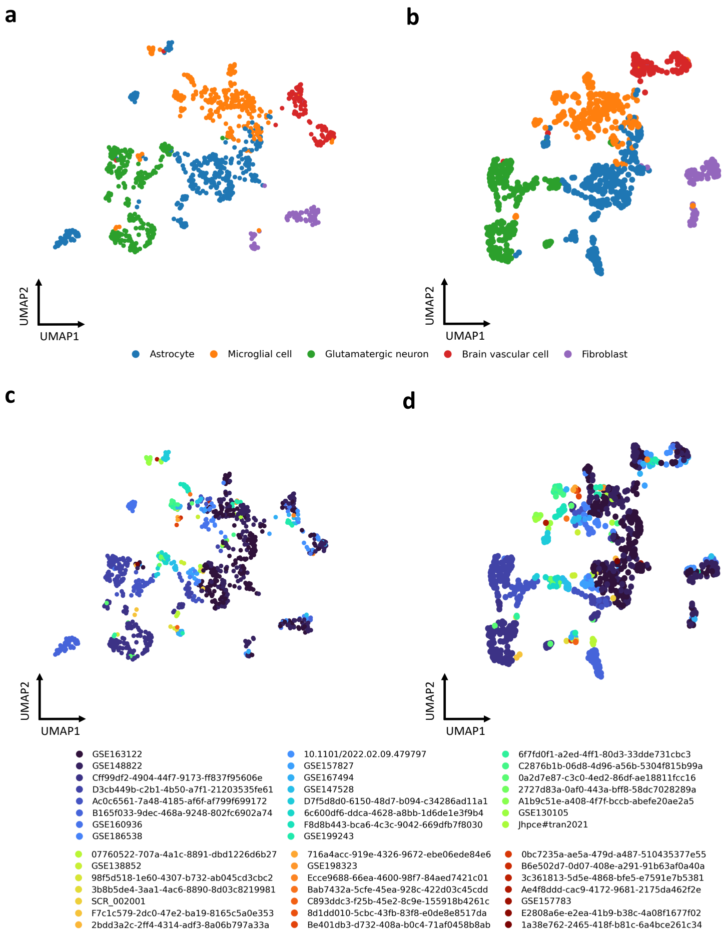

Fig. S1: UMAP embeddings of AD cells before and after ComBat correction. **(a)** Cell-type distribution (pre-ComBat); **(b)** cell-type distribution (post-ComBat); **(c)** data-source distribution (pre-ComBat); **(d)** data-source distribution (post-ComBat).

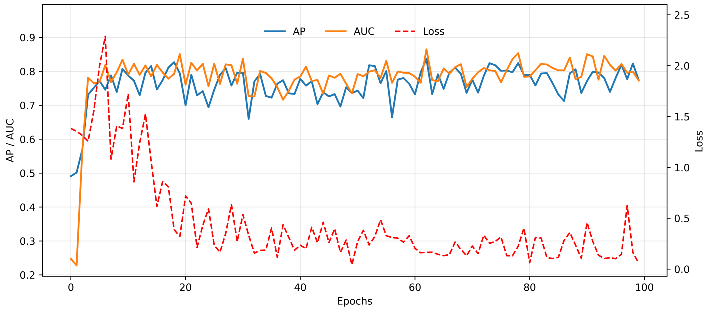

Fig. S2: Experimental results of CellTOSG-FM pretraining

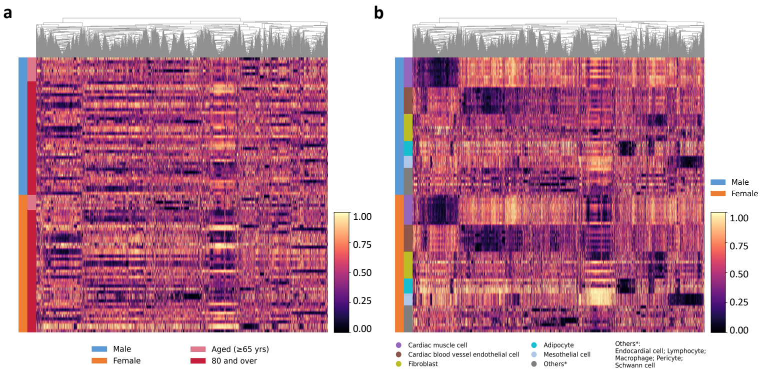

Fig. S3: Cell embeddings learned by CellTOSG-FM for sex classification tasks on AF dataset. (a) Cell sex classification task on AF dataset stratified by age groups. **(b)** Cell sex classification task on AF dataset stratified by cell types.
